# Supplementary material for: Genomic surveillance of SARS-CoV-2 Spike gene by sanger sequencing
Source: PLoS One. 2022 Jan 20;17(1):e0262170. doi: 10.1371/journal.pone.0262170 (PMC8775319; doi:10.1371/journal.pone.0262170)
Supplement: S1 File — (PDF) [file pone.0262170.s001.pdf]

Sanger sequencing of SARS-CoV-2 Spike protein

Tiago S. Salles*[1], Andrea Cony Cavalcanti*[2], Fabio Burack da Costa[1], Renata Campos Azevedo[1]

[1]Laboratório de Interação Vírus-Célula, Departamento de Virologia, Inst. de Microbiologia Paulo de Góes, Universidade Federal do Rio de Janeiro, Rio de Janeiro, Brazil;

[2]Laboratório Central de Saúde Pública Noel Nutels - LACEN-RJ, Rio de Janeiro, Brazil

dx.doi.org/10.17504/protocols.io.bx6kprcw

fabio_burack

ABSTRACT

The SARS-CoV-2 responsible for the ongoing COVID pandemic reveals particular evolutionary dynamics and an extensive polymorphism, mainly in Spike protein. Monitoring the S protein mutations is crucial for successful controlling measures and detect variants that can evade vaccine immunity. Even after the costs reduction imposed by the pandemic, the new generation sequencing methodologies remain unavailable to many scientific groups. Therefore, to support the urgent surveillance of SARS-CoV-2 S protein, this work describes a protocol for complete nucleotide sequencing of the S protein using the Sanger technique. Thus, any laboratory with experience in sequencing can adopt this protocol.

DOI

dx.doi.org/10.17504/protocols.io.bx6kprcw

PROTOCOL CITATION

Tiago S. Salles*, Andrea Cony Cavalcanti*, Fabio Burack da Costa, Renata Campos Azevedo . Sanger sequencing of SARS-CoV-2 Spike protein. **protocols.io** https://dx.doi.org/10.17504/protocols.io.bx6kprcw

KEYWORDS

Sanger sequencing, SARS-CoV-2, Spike protein

LICENSE

This is an open access protocol distributed under the terms of the Creative Commons Attribution License, which permits unrestricted use, distribution, and reproduction in any medium, provided the original author and source are credited

CREATED

Sep 13, 2021

LAST MODIFIED

Oct 06, 2021

PROTOCOL INTEGER ID

53164

GUIDELINES

This protocol works well with recent extracted RNA samples, ideally with ct values lower than 20.

MATERIALS TEXT

- Thermal cycler
- PCR tubes 0.2mL
- Filter pipette tips: 1-10µL+ 10-100µL
- Micropipettes: 1-10µL+ 10-100µL
- Superscript III one-step RT-PCR kit (Invitrogen, Carlsbad, CA, USA)
- Primers
- Nuclease free water
- Extracted RNA from SARS-CoV-2 positive samples with low ct values
- Agarose
- TAE buffer (Tris-acetate-EDTA)
- LGC biotecnologia - Blue Green loading Dye I
- Horizontal Electrophoresis cube
- UV Transilluminator
- Nanodrop spectrophotometer

1 RT-PCR

- Program the thermal cycler before setting up the reaction. The thermal cycler should be preheated to 45–60°C.
- Keep all components, reaction mixes, and samples on ice. After preparation of the samples, transfer them to the preheated thermal cycler and immediately start the RT–PCR program.
- Reaction mix should be prepared according to table 1.

A	B	C
Component	Volume	Final concentration
2X Reaction Mix	12.5 µL	1x
Sense primer (10 µM)	1.75 µL	0.7 µM
Anti-sense primer (10 µM)	1.75 µL	0.7 µM
SuperScript™ III RT/Platinum™ Taq High Fidelity Enzyme Mix[1]	0.5 µL	
Template RNA	5 µL	
Nuclease free water	to 25 µL	

Table 1 - RT-PCR master mix

- **RT-PCR cycle is described below**:

60°C 1 min |
50°C 45 min | Reverse Transcription | 1 cycle
94°C 2 min |

95 °C 15 s Denaturation |
53 °C 30 s Annealing | 40 cycles
68 °C 60 s Extension |

68 °C 7 min Final extention | 1 cycle
4 °C ∞

- **Primers used**:

Primer		Sequence	Position	Melting temperature	Size
P1	forward	GTTTGTTTTTCTTGTTTTATT	(21551-21574)	43.5 ºC	923bp
P1	reverse	ACAGTGAAGGATTTCAACGTACAC	(22450-22474)	55.3 ºC	
P2	forward	CGTGATCTCCCTCAGGGTTTT	(22190-22211)	56.8 ºC	620bp
P2	reverse	TCAGCAATCTTTCCAGTTTGCC	(22810-22832)	56.1 ºC	
P3	forward	GTAATTAGAGGTGATGAAGTCAGA	(22751-22775)	51.8 ºC	892bp
P3	reverse	ACATAGTGTAGGCAATGATGGA	(23621-23643)	53.6 ºC	
P4	forward	CTTGGCGTGTTTATTCTACAG	(23445-23466)	51.4 ºC	979bp
P4	reverse	GCTTGTGCATTTTGGTTGACC	(24403-24424)	55.6 ºC	
P5	forward	AGACTCACTTTCTTCCACAGCA	(24355-24377)	56.1 ºC	342bp
P5	reverse	AGATGATAGCCCTTTCCACA	(24699-24719)	53.3 ºC	
P6	forward	TTCTGCTAATCTTGCTGCTACT	(24610-24632)	54.0 ºC	766bp
P6	reverse	GTTTATGTGTAATGTAATTTGACTCC	(25348-25372)	50.7 ºC	
P7	forward	TAGAGAAAACAACAGAGTT	(21492-21511)	45.6 ºC	712bp
P7	reverse	TGAGGGAGATCACGCACTAA	(22184-22204)	55.4 ºC	
P8	forward	TTCTGCTAATCTTGCTGCTACT	(24610-24632)	54.0 ºC	825bp
P8	reverse	CCTTGCTTCAAAGTTACAGTTCCA	(25409-25433)	55.6 ºC	

2 Agarose gel Electrophoresis

- Dissolve agarose in 1.X TAE Buffer (40 mM Tris-acetate, 1 mM EDTA) to a final concentration of 1.5% agarose.
- Heat the solution in a microwave and let it cool at room temperature.
- Apply the agarose gel to the casting tray with the appropriate well comb and let it solidify.
- Mix 5 µL of PCR product with 5µL of loading buffer and 1µL Blue Green Loading dye I (LGC

Biotecnologia), apply it to the gel, and run the electrophoresis at 120V.
- Visualize the gel with UV Transilluminator

3 Preparing Samples for sequencing

- Sequencing procedures are performed according to BigDye™ Terminator v3.1 Cycle Sequencing Kit (Applied Biosystems) guidelines.
- Measure DNA Concentration with a nanodrop spectrophotometer
- Dilute template to 200 ng/µl with nuclease-free water
- Dilute primers to 1 µM with nuclease-free water. Only one primer is used for each sequencing reaction, leading to two reactions per sample. Each reaction will need 1µl of diluted primer.
- Label and ship the samples and primers according to the sequencing service provider guidelines.

sequencing reaction:
- BigDye™ Terminator 3.1 Ready Reaction Mix - 8 µL
- primer (1 µM) - 6.4 µL
- Template (200ng) - 2 µL
- Deionized water (RNase/DNase-free) to - 20 µL

sequencing cycle:

96°C 1 min Incubation | 1 cycle

96 °C 10 s Denaturation |
50 °C 05 s Annealing | 25 cycles
60 °C 4 min Extension |

4 °C ∞ | 1 cycle

4 Sequence analysis

- Upon receiving the electropherograms, edit them with proper programs like chromas or Bioedit.
- For better editing accuracy, align the forward sequence with the reverse complement of the reverse sequence.
- Use BLAST search to confirm if the sequenced product corresponds to the desired target.
- Create contigs by aligning the planned overlaps between each target fragment and form one consensus sequence covering the full ORF of SARS-CoV-2 Spike protein
- Edited sequences can now be analyzed with the CoVsurver mutations app, provided by GISAID, to trace the mutation patterns of each sample and study their effects on protein structure.
- Sequences can also be deposited in the GISAID database of SARS-CoV-2.
