# Supplementary material for: Genomic surveillance of SARS-CoV-2 Spike gene by sanger sequencing
Source: PLoS One. 2022 Jan 20;17(1):e0262170. doi: 10.1371/journal.pone.0262170 (PMC8775319; doi:10.1371/journal.pone.0262170)
Supplement: S2 File — (PDF) [file pone.0262170.s002.pdf]

S2 File. Map of primer pairs in VOCs sequences.

```
                      10         20         30         40         50
              ....|....| ....|....| ....|....| ....|....| ....|....|
Wuhan-Hu-1    ATGTTTGTTT TTCTTGTTTT ATTGCCACTA GTCTCTAGTC AGTGTGTTAA
B.1.1.7       ATGTTTGTTT TTCTTGTTTT ATTGCCACTA GTCTCTAGTC AGTGTGTTAA
B.1.351       ATGTTTGTTT TTCTTGTTTT ATTGCCACTA GTCTCTAGTC AGTGTGTTAA
P.1           ATGTTTGTTT TTCTTGTTTT ATTGCCACTA GTCTCTAGTC AGTGTGTTAA
B.1.617       ATGTTTGTTT TTCTTGTTTT ATTGCCACTA GTCTCTAGTC AGTGTGTTAA
PRIMER 1F     --GTTTGTTT TTCTTGTTTT ATT------- ---------- ----------
PRIMER 1R     ---------- ---------- ---------- ---------- ----------
PRIMER 2F     ---------- ---------- ---------- ---------- ----------
PRIMER 2R     ---------- ---------- ---------- ---------- ----------
PRIMER 3F     ---------- ---------- ---------- ---------- ----------
PRIMER 3R     ---------- ---------- ---------- ---------- ----------
PRIMER 4F     ---------- ---------- ---------- ---------- ----------
PRIMER 4R     ---------- ---------- ---------- ---------- ----------
PRIMER 5F     ---------- ---------- ---------- ---------- ----------
PRIMER 5R     ---------- ---------- ---------- ---------- ----------
PRIMER 6F     ---------- ---------- ---------- ---------- ----------
PRIMER 6R     ---------- ---------- ---------- ---------- ----------
PRIMER 7R     ---------- ---------- ---------- ---------- ----------
PRIMER 8F     ---------- ---------- ---------- ---------- ----------

                      60         70         80         90        100
              ....|....| ....|....| ....|....| ....|....| ....|....|
Wuhan-Hu-1    TCTTACAACC AGAACTCAAT TACCCCCTGC ATACACTAAT TCTTTCACAC
B.1.1.7       TCTTACAACC AGAACTCAAT TACCCCCTGC ATACACTAAT TCTTTCACAC
B.1.351       TCTTACAACC AGAACTCAAT TACCCCCTGC ATACACTAAT TCTTTCACAC
P.1           TTTTACAAAC AGAACTCAAT TACCCTCTGC ATACACTAAT TCTTTCACAC
B.1.617       TCTTAGAACC AGAACTCAAT TACCCCCTGC ATACACTAAT TCTTTCACAC
PRIMER 1F     ---------- ---------- ---------- ---------- ----------
PRIMER 1R     ---------- ---------- ---------- ---------- ----------
PRIMER 2F     ---------- ---------- ---------- ---------- ----------
PRIMER 2R     ---------- ---------- ---------- ---------- ----------
PRIMER 3F     ---------- ---------- ---------- ---------- ----------
PRIMER 3R     ---------- ---------- ---------- ---------- ----------
PRIMER 4F     ---------- ---------- ---------- ---------- ----------
PRIMER 4R     ---------- ---------- ---------- ---------- ----------
PRIMER 5F     ---------- ---------- ---------- ---------- ----------
PRIMER 5R     ---------- ---------- ---------- ---------- ----------
PRIMER 6F     ---------- ---------- ---------- ---------- ----------
PRIMER 6R     ---------- ---------- ---------- ---------- ----------
PRIMER 7R     ---------- ---------- ---------- ---------- ----------
PRIMER 8F     ---------- ---------- ---------- ---------- ----------

                     110        120        130        140        150
              ....|....| ....|....| ....|....| ....|....| ....|....|
Wuhan-Hu-1    GTGGTGTTTA TTACCCTGAC AAAGTTTTCA GATCCTCAGT TTTACATTCA
B.1.1.7       GTGGTGTTTA TTACCCTGAC AAAGTTTTCA GATCCTCAGT TTTACATTCA
B.1.351       GTGGTGTTTA TTACCCTGAC AAAGTTTTCA GATCCTCAGT TTTACATTCA
P.1           GTGGTGTTTA TTACCCTGAC AAAGTTTTCA GATCCTCAGT TTTACATTCA
B.1.617       GTGGTGTTTA TTACCCTGAC AAAGTTTTCA GATCCTCAGT TTTACATTCA
PRIMER 1F     ---------- ---------- ---------- ---------- ----------
PRIMER 1R     ---------- ---------- ---------- ---------- ----------
PRIMER 2F     ---------- ---------- ---------- ---------- ----------
PRIMER 2R     ---------- ---------- ---------- ---------- ----------
PRIMER 3F     ---------- ---------- ---------- ---------- ----------
PRIMER 3R     ---------- ---------- ---------- ---------- ----------
```

```
PRIMER 4F    ---------- ---------- ---------- ---------- ----------
PRIMER 4R    ---------- ---------- ---------- ---------- ----------
PRIMER 5F    ---------- ---------- ---------- ---------- ----------
PRIMER 5R    ---------- ---------- ---------- ---------- ----------
PRIMER 6F    ---------- ---------- ---------- ---------- ----------
PRIMER 6R    ---------- ---------- ---------- ---------- ----------
PRIMER 7R    ---------- ---------- ---------- ---------- ----------
PRIMER 8F    ---------- ---------- ---------- ---------- ----------

                    160        170        180        190        200
             ....|....| ....|....| ....|....| ....|....| ....|....|
Wuhan-Hu-1   ACTCAGGACT TGTTCTTACC TTTCTTTTCC AATGTTACTT GGTTCCATGC
B.1.1.7      ACTCAGGACT TGTTCTTACC TTTCTTTTCC AATGTTACTT GGTTCCATGC
B.1.351      ACTCAGGACT TGTTCTTACC TTTCTTTTCC AATGTTACTT GGTTCCATGC
P.1          ACTCAGGACT TGTTCTTACC TTTCTTTTCC AATGTTACTT GGTTCCATGC
B.1.617      ACTCAGGACT TGTTCTTACC TTTCTTTTCC AATGTTACTT GGTTCCATGC
PRIMER 1F    ---------- ---------- ---------- ---------- ----------
PRIMER 1R    ---------- ---------- ---------- ---------- ----------
PRIMER 2F    ---------- ---------- ---------- ---------- ----------
PRIMER 2R    ---------- ---------- ---------- ---------- ----------
PRIMER 3F    ---------- ---------- ---------- ---------- ----------
PRIMER 3R    ---------- ---------- ---------- ---------- ----------
PRIMER 4F    ---------- ---------- ---------- ---------- ----------
PRIMER 4R    ---------- ---------- ---------- ---------- ----------
PRIMER 5F    ---------- ---------- ---------- ---------- ----------
PRIMER 5R    ---------- ---------- ---------- ---------- ----------
PRIMER 6F    ---------- ---------- ---------- ---------- ----------
PRIMER 6R    ---------- ---------- ---------- ---------- ----------
PRIMER 7R    ---------- ---------- ---------- ---------- ----------
PRIMER 8F    ---------- ---------- ---------- ---------- ----------

                    210        220        230        240        250
             ....|....| ....|....| ....|....| ....|....| ....|....|
Wuhan-Hu-1   TATACATGTC TCTGGGACCA ATGGTACTAA GAGGTTTGAT AACCCTGTCC
B.1.1.7      TA------TC TCTGGGACCA ATGGTACTAA GAGGTTTGAT AACCCTGTCC
B.1.351      TATACATGTC TCTGGGACCA ATGGTACTAA GAGGTTTGCT AACCCTGTCC
P.1          TATACATGTC TCTGGGACCA ATGGTACTAA GAGGTTTGAT AACCCTGTCC
B.1.617      TATACATGTC TCTGGGACCA ATGGTACTAA GAGGTTTGAT AACCCTGTCC
PRIMER 1F    ---------- ---------- ---------- ---------- ----------
PRIMER 1R    ---------- ---------- ---------- ---------- ----------
PRIMER 2F    ---------- ---------- ---------- ---------- ----------
PRIMER 2R    ---------- ---------- ---------- ---------- ----------
PRIMER 3F    ---------- ---------- ---------- ---------- ----------
PRIMER 3R    ---------- ---------- ---------- ---------- ----------
PRIMER 4F    ---------- ---------- ---------- ---------- ----------
PRIMER 4R    ---------- ---------- ---------- ---------- ----------
PRIMER 5F    ---------- ---------- ---------- ---------- ----------
PRIMER 5R    ---------- ---------- ---------- ---------- ----------
PRIMER 6F    ---------- ---------- ---------- ---------- ----------
PRIMER 6R    ---------- ---------- ---------- ---------- ----------
PRIMER 7R    ---------- ---------- ---------- ---------- ----------
PRIMER 8F    ---------- ---------- ---------- ---------- ----------

                    260        270        280        290        300
             ....|....| ....|....| ....|....| ....|....| ....|....|
Wuhan-Hu-1   TACCATTTAA TGATGGTGTT TATTTTGCTT CCACTGAGAA GTCTAACATA
B.1.1.7      TACCATTTAA TGATGGTGTT TATTTTGCTT CCACTGAGAA GTCTAACATA
B.1.351      TACCATTTAA TGATGGTGTT TATTTTGCTT CCACTGAGAA GTCTAACATA
P.1          TACCATTTAA TGATGGTGTT TATTTTGCTT CCACTGAGAA GTCTAACATA
B.1.617      TACCATTTAA TGATGGTGTT TATTTTGCTT CCACTGAGAA GTCTAACATA
PRIMER 1F    ---------- ---------- ---------- ---------- ----------
PRIMER 1R    ---------- ---------- ---------- ---------- ----------
```

```
PRIMER 2F    ---------- ---------- ---------- ---------- ----------
PRIMER 2R    ---------- ---------- ---------- ---------- ----------
PRIMER 3F    ---------- ---------- ---------- ---------- ----------
PRIMER 3R    ---------- ---------- ---------- ---------- ----------
PRIMER 4F    ---------- ---------- ---------- ---------- ----------
PRIMER 4R    ---------- ---------- ---------- ---------- ----------
PRIMER 5F    ---------- ---------- ---------- ---------- ----------
PRIMER 5R    ---------- ---------- ---------- ---------- ----------
PRIMER 6F    ---------- ---------- ---------- ---------- ----------
PRIMER 6R    ---------- ---------- ---------- ---------- ----------
PRIMER 7R    ---------- ---------- ---------- ---------- ----------
PRIMER 8F    ---------- ---------- ---------- ---------- ----------

                    310        320        330        340        350
             ....|....| ....|....| ....|....| ....|....| ....|....|
Wuhan-Hu-1   ATAAGAGGCT GGATTTTTGG TACTACTTTA GATTCGAAGA CCCAGTCCCT
B.1.1.7      ATAAGAGGCT GGATTTTTGG TACTACTTTA GATTCGAAGA CCCAGTCCCT
B.1.351      ATAAGAGGCT GGATTTTTGG TACTACTTTA GATTCGAAGA CCCAGTCCCT
P.1          ATAAGAGGCT GGATTTTTGG TACTACTTTA GATTCGAAGA CCCAGTCCCT
B.1.617      ATAAGAGGCT GGATTTTTGG TACTACTTTA GATTCGAAGA CCCAGTCCCT
PRIMER 1F    ---------- ---------- ---------- ---------- ----------
PRIMER 1R    ---------- ---------- ---------- ---------- ----------
PRIMER 2F    ---------- ---------- ---------- ---------- ----------
PRIMER 2R    ---------- ---------- ---------- ---------- ----------
PRIMER 3F    ---------- ---------- ---------- ---------- ----------
PRIMER 3R    ---------- ---------- ---------- ---------- ----------
PRIMER 4F    ---------- ---------- ---------- ---------- ----------
PRIMER 4R    ---------- ---------- ---------- ---------- ----------
PRIMER 5F    ---------- ---------- ---------- ---------- ----------
PRIMER 5R    ---------- ---------- ---------- ---------- ----------
PRIMER 6F    ---------- ---------- ---------- ---------- ----------
PRIMER 6R    ---------- ---------- ---------- ---------- ----------
PRIMER 7R    ---------- ---------- ---------- ---------- ----------
PRIMER 8F    ---------- ---------- ---------- ---------- ----------

                    360        370        380        390        400
             ....|....| ....|....| ....|....| ....|....| ....|....|
Wuhan-Hu-1   ACTTATTGTT AATAACGCTA CTAATGTTGT TATTAAAGTC TGTGAATTTC
B.1.1.7      ACTTATTGTT AATAACGCTA CTAATGTTGT TATTAAAGTC TGTGAATTTC
B.1.351      ACTTATTGTT AATAACGCTA CTAATGTTGT TATTAAAGTC TGTGAATTTC
P.1          ACTTATTGTT AATAACGCTA CTAATGTTGT TATTAAAGTC TGTGAATTTC
B.1.617      ACTTATTGTT AATAACGCTA CTAATGTTGT TATTAAAGTC TGTGAATTTC
PRIMER 1F    ---------- ---------- ---------- ---------- ----------
PRIMER 1R    ---------- ---------- ---------- ---------- ----------
PRIMER 2F    ---------- ---------- ---------- ---------- ----------
PRIMER 2R    ---------- ---------- ---------- ---------- ----------
PRIMER 3F    ---------- ---------- ---------- ---------- ----------
PRIMER 3R    ---------- ---------- ---------- ---------- ----------
PRIMER 4F    ---------- ---------- ---------- ---------- ----------
PRIMER 4R    ---------- ---------- ---------- ---------- ----------
PRIMER 5F    ---------- ---------- ---------- ---------- ----------
PRIMER 5R    ---------- ---------- ---------- ---------- ----------
PRIMER 6F    ---------- ---------- ---------- ---------- ----------
PRIMER 6R    ---------- ---------- ---------- ---------- ----------
PRIMER 7R    ---------- ---------- ---------- ---------- ----------
PRIMER 8F    ---------- ---------- ---------- ---------- ----------

                    410        420        430        440        450
             ....|....| ....|....| ....|....| ....|....| ....|....|
Wuhan-Hu-1   AATTTTGTAA TGATCCATTT TTGGGTGTTT ATTACCACAA AAACAACAAA
B.1.1.7      AATTTTGTAA TGATCCATTT TTGGGTGTT- --TACCACAA AAACAACAAA
B.1.351      AATTTTGTAA TGATCCATTT TTGGGTGTTT ATTACCACAA AAACAACAAA
```

```
P.1           AATTTTGTAA TTATCCATTT TTGGGTGTTT ATTACCACAA AAACAACAAA
B.1.617       AATTTTGTAA TGATCCATTT TTGGGTGTTT ATTACCACAA AAACAACAAA
PRIMER 1F     ---------- ---------- ---------- ---------- ----------
PRIMER 1R     ---------- ---------- ---------- ---------- ----------
PRIMER 2F     ---------- ---------- ---------- ---------- ----------
PRIMER 2R     ---------- ---------- ---------- ---------- ----------
PRIMER 3F     ---------- ---------- ---------- ---------- ----------
PRIMER 3R     ---------- ---------- ---------- ---------- ----------
PRIMER 4F     ---------- ---------- ---------- ---------- ----------
PRIMER 4R     ---------- ---------- ---------- ---------- ----------
PRIMER 5F     ---------- ---------- ---------- ---------- ----------
PRIMER 5R     ---------- ---------- ---------- ---------- ----------
PRIMER 6F     ---------- ---------- ---------- ---------- ----------
PRIMER 6R     ---------- ---------- ---------- ---------- ----------
PRIMER 7R     ---------- ---------- ---------- ---------- ----------
PRIMER 8F     ---------- ---------- ---------- ---------- ----------

                     460        470        480        490        500
              ....|....| ....|....| ....|....| ....|....| ....|....|
Wuhan-Hu-1    AGTTGGATGG AAAGTGAGTT CAGAGTTTAT TCTAGTGCGA ATAATTGCAC
B.1.1.7       AGTTGGATGG AAAGTGAGTT CAGAGTTTAT TCTAGTGCGA ATAATTGCAC
B.1.351       AGTTGGATGG AAAGTGAGTT CAGAGTTTAT TCTAGTGCGA ATAATTGCAC
P.1           AGTTGGATGG AAAGTGAGTT CAGAGTTTAT TCTAGTGCGA ATAATTGCAC
B.1.617       AGTTGGATGG AAAGTG---- --GAGTTTAT TCTAGTGCGA ATAATTGCAC
PRIMER 1F     ---------- ---------- ---------- ---------- ----------
PRIMER 1R     ---------- ---------- ---------- ---------- ----------
PRIMER 2F     ---------- ---------- ---------- ---------- ----------
PRIMER 2R     ---------- ---------- ---------- ---------- ----------
PRIMER 3F     ---------- ---------- ---------- ---------- ----------
PRIMER 3R     ---------- ---------- ---------- ---------- ----------
PRIMER 4F     ---------- ---------- ---------- ---------- ----------
PRIMER 4R     ---------- ---------- ---------- ---------- ----------
PRIMER 5F     ---------- ---------- ---------- ---------- ----------
PRIMER 5R     ---------- ---------- ---------- ---------- ----------
PRIMER 6F     ---------- ---------- ---------- ---------- ----------
PRIMER 6R     ---------- ---------- ---------- ---------- ----------
PRIMER 7R     ---------- ---------- ---------- ---------- ----------
PRIMER 8F     ---------- ---------- ---------- ---------- ----------

                     510        520        530        540        550
              ....|....| ....|....| ....|....| ....|....| ....|....|
Wuhan-Hu-1    TTTTGAATAT GTCTCTCAGC CTTTTCTTAT GGACCTTGAA GGAAAACAGG
B.1.1.7       TTTTGAATAT GTCTCTCAGC CTTTTCTTAT GGACCTTGAA GGAAAACAGG
B.1.351       TTTTGAATAT GTCTCTCAGC CTTTTCTTAT GGACCTTGAA GGAAAACAGG
P.1           TTTTGAATAT GTCTCTCAGC CTTTTCTTAT GGACCTTGAA GGAAAACAGG
B.1.617       TTTTGAATAT GTCTCTCAGC CTTTTCTTAT GGACCTTGAA GGAAAACAGG
PRIMER 1F     ---------- ---------- ---------- ---------- ----------
PRIMER 1R     ---------- ---------- ---------- ---------- ----------
PRIMER 2F     ---------- ---------- ---------- ---------- ----------
PRIMER 2R     ---------- ---------- ---------- ---------- ----------
PRIMER 3F     ---------- ---------- ---------- ---------- ----------
PRIMER 3R     ---------- ---------- ---------- ---------- ----------
PRIMER 4F     ---------- ---------- ---------- ---------- ----------
PRIMER 4R     ---------- ---------- ---------- ---------- ----------
PRIMER 5F     ---------- ---------- ---------- ---------- ----------
PRIMER 5R     ---------- ---------- ---------- ---------- ----------
PRIMER 6F     ---------- ---------- ---------- ---------- ----------
PRIMER 6R     ---------- ---------- ---------- ---------- ----------
PRIMER 7R     ---------- ---------- ---------- ---------- ----------
PRIMER 8F     ---------- ---------- ---------- ---------- ----------

                     560        570        580        590        600
```

```
             ....|....| ....|....| ....|....| ....|....| ....|....|
Wuhan-Hu-1   GTAATTTCAA AAATCTTAGG GAATTTGTGT TTAAGAATAT TGATGGTTAT
B.1.1.7      GTAATTTCAA AAATCTTAGG GAATTTGTGT TTAAGAATAT TGATGGTTAT
B.1.351      GTAATTTCAA AAATCTTAGG GAATTTGTGT TTAAGAATAT TGATGGTTAT
P.1          GTAATTTCAA AAATCTTAGT GAATTTGTGT TTAAGAATAT TGATGGTTAT
B.1.617      GTAATTTCAA AAATCTTAGG GAATTTGTGT TTAAGAATAT TGATGGTTAT
PRIMER 1F    ---------- ---------- ---------- ---------- ----------
PRIMER 1R    ---------- ---------- ---------- ---------- ----------
PRIMER 2F    ---------- ---------- ---------- ---------- ----------
PRIMER 2R    ---------- ---------- ---------- ---------- ----------
PRIMER 3F    ---------- ---------- ---------- ---------- ----------
PRIMER 3R    ---------- ---------- ---------- ---------- ----------
PRIMER 4F    ---------- ---------- ---------- ---------- ----------
PRIMER 4R    ---------- ---------- ---------- ---------- ----------
PRIMER 5F    ---------- ---------- ---------- ---------- ----------
PRIMER 5R    ---------- ---------- ---------- ---------- ----------
PRIMER 6F    ---------- ---------- ---------- ---------- ----------
PRIMER 6R    ---------- ---------- ---------- ---------- ----------
PRIMER 7R    ---------- ---------- ---------- ---------- ----------
PRIMER 8F    ---------- ---------- ---------- ---------- ----------

                    610        620        630        640        650
             ....|....| ....|....| ....|....| ....|....| ....|....|
Wuhan-Hu-1   TTTAAAATAT ATTCTAAGCA CACGCCTATT AATTTAGTGC GTGATCTCCC
B.1.1.7      TTTAAAATAT ATTCTAAGCA CACGCCTATT AATTTAGTGC GTGATCTCCC
B.1.351      TTTAAAATAT ATTCTAAGCA CACGCCTATT AATTTAGTGC GTGATCTCCC
P.1          TTTAAAATAT ATTCTAAGCA CACGCCTATT AATTTAGTGC GTGATCTCCC
B.1.617      TTTAAAATAT ATTCTAAGCA CACGCCTATT AATTTAGTGC GTGATCTCCC
PRIMER 1F    ---------- ---------- ---------- ---------- ----------
PRIMER 1R    ---------- ---------- ---------- ---------- ----------
PRIMER 2F    ---------- ---------- ---------- ---------C GTGATCTCCC
PRIMER 2R    ---------- ---------- ---------- ---------- ----------
PRIMER 3F    ---------- ---------- ---------- ---------- ----------
PRIMER 3R    ---------- ---------- ---------- ---------- ----------
PRIMER 4F    ---------- ---------- ---------- ---------- ----------
PRIMER 4R    ---------- ---------- ---------- ---------- ----------
PRIMER 5F    ---------- ---------- ---------- ---------- ----------
PRIMER 5R    ---------- ---------- ---------- ---------- ----------
PRIMER 6F    ---------- ---------- ---------- ---------- ----------
PRIMER 6R    ---------- ---------- ---------- ---------- ----------
PRIMER 7R    ---------- ---------- ---------- ---TTAGTGC GTGATCTCCC
PRIMER 8F    ---------- ---------- ---------- ---------- ----------

                    660        670        680        690        700
             ....|....| ....|....| ....|....| ....|....| ....|....|
Wuhan-Hu-1   TCAGGGTTTT TCGGCTTTAG AACCATTGGT AGATTTGCCA ATAGGTATTA
B.1.1.7      TCAGGGTTTT TCGGCTTTAG AACCATTGGT AGATTTGCCA ATAGGTATTA
B.1.351      TCAGGGTTTT TCGGCTTTAG AACCATTGGT AGATTTGCCA ATAGGTATTA
P.1          TCAGGGTTTT TCGGCTTTAG AACCATTGGT AGATTTGCCA ATAGGTATTA
B.1.617      TCAGGGTTTT TCGGTTTTAG AACCATTGGT AGATTTGCCA ATAGGTATTA
PRIMER 1F    ---------- ---------- ---------- ---------- ----------
PRIMER 1R    ---------- ---------- ---------- ---------- ----------
PRIMER 2F    TCAGGGTTTT ---------- ---------- ---------- ----------
PRIMER 2R    ---------- ---------- ---------- ---------- ----------
PRIMER 3F    ---------- ---------- ---------- ---------- ----------
PRIMER 3R    ---------- ---------- ---------- ---------- ----------
PRIMER 4F    ---------- ---------- ---------- ---------- ----------
PRIMER 4R    ---------- ---------- ---------- ---------- ----------
PRIMER 5F    ---------- ---------- ---------- ---------- ----------
PRIMER 5R    ---------- ---------- ---------- ---------- ----------
PRIMER 6F    ---------- ---------- ---------- ---------- ----------
PRIMER 6R    ---------- ---------- ---------- ---------- ----------
```

```
PRIMER 7R    TCA------- ---------- ---------- ---------- ----------
PRIMER 8F    ---------- ---------- ---------- ---------- ----------

                      710        720        730        740        750
             ....|....| ....|....| ....|....| ....|....| ....|....|
Wuhan-Hu-1   ACATCACTAG GTTTCAAACT TTACTTGCTT TACATAGAAG TTATTTGACT
B.1.1.7      ACATCACTAG GTTTCAAACT TTACTTGCTT TACATAGAAG TTATTTGACT
B.1.351      ACATCACTAG GTTTCAAACT TTACTTGCTT TACATAGAAG TTATTTGACT
P.1          ACATCACTAG GTTTCAAACT TTACTTGCTT TACATAGAAG TTATTTGACT
B.1.617      ACATCACTAG GTTTCAAACT TTACTTGCTT TACATAGAAG TTATTTGACT
PRIMER 1F    ---------- ---------- ---------- ---------- ----------
PRIMER 1R    ---------- ---------- ---------- ---------- ----------
PRIMER 2F    ---------- ---------- ---------- ---------- ----------
PRIMER 2R    ---------- ---------- ---------- ---------- ----------
PRIMER 3F    ---------- ---------- ---------- ---------- ----------
PRIMER 3R    ---------- ---------- ---------- ---------- ----------
PRIMER 4F    ---------- ---------- ---------- ---------- ----------
PRIMER 4R    ---------- ---------- ---------- ---------- ----------
PRIMER 5F    ---------- ---------- ---------- ---------- ----------
PRIMER 5R    ---------- ---------- ---------- ---------- ----------
PRIMER 6F    ---------- ---------- ---------- ---------- ----------
PRIMER 6R    ---------- ---------- ---------- ---------- ----------
PRIMER 7R    ---------- ---------- ---------- ---------- ----------
PRIMER 8F    ---------- ---------- ---------- ---------- ----------

                      760        770        780        790        800
             ....|....| ....|....| ....|....| ....|....| ....|....|
Wuhan-Hu-1   CCTGGTGATT CTTCTTCAGG TTGGACAGCT GGTGCTGCAG CTTATTATGT
B.1.1.7      CCTGGTGATT CTTCTTCAGG TTGGACAGCT GGTGCTGCAG CTTATTATGT
B.1.351      CCTGGTGATT CTTCTTCAGG TTGGACAGCT GGTGCTGCAG CTTATTATGT
P.1          CCTGGTGATT CTTCTTCAGG TTGGACAGCT GGTGCTGCAG CTTATTATGT
B.1.617      CCTGGTGATT CTTCTTCAGG TTGGACAGCT GGTGCTGCAG CTTATTATGT
PRIMER 1F    ---------- ---------- ---------- ---------- ----------
PRIMER 1R    ---------- ---------- ---------- ---------- ----------
PRIMER 2F    ---------- ---------- ---------- ---------- ----------
PRIMER 2R    ---------- ---------- ---------- ---------- ----------
PRIMER 3F    ---------- ---------- ---------- ---------- ----------
PRIMER 3R    ---------- ---------- ---------- ---------- ----------
PRIMER 4F    ---------- ---------- ---------- ---------- ----------
PRIMER 4R    ---------- ---------- ---------- ---------- ----------
PRIMER 5F    ---------- ---------- ---------- ---------- ----------
PRIMER 5R    ---------- ---------- ---------- ---------- ----------
PRIMER 6F    ---------- ---------- ---------- ---------- ----------
PRIMER 6R    ---------- ---------- ---------- ---------- ----------
PRIMER 7R    ---------- ---------- ---------- ---------- ----------
PRIMER 8F    ---------- ---------- ---------- ---------- ----------

                      810        820        830        840        850
             ....|....| ....|....| ....|....| ....|....| ....|....|
Wuhan-Hu-1   GGGTTATCTT CAACCTAGGA CTTTTCTATT AAAATATAAT GAAAATGGAA
B.1.1.7      GGGTTATCTT CAACCTAGGA CTTTTCTATT AAAATATAAT GAAAATGGAA
B.1.351      GGGTTATCTT CAACCTAGGA CTTTTCTATT AAAATATAAT GAAAATGGAA
P.1          GGGTTATCTT CAACCTAGGA CTTTTCTATT AAAATATAAT GAAAATGGAA
B.1.617      GGGTTATCTT CAACCTAGGA CTTTTCTATT AAAATATAAT GAAAATGGAA
PRIMER 1F    ---------- ---------- ---------- ---------- ----------
PRIMER 1R    ---------- ---------- ---------- ---------- ----------
PRIMER 2F    ---------- ---------- ---------- ---------- ----------
PRIMER 2R    ---------- ---------- ---------- ---------- ----------
PRIMER 3F    ---------- ---------- ---------- ---------- ----------
PRIMER 3R    ---------- ---------- ---------- ---------- ----------
PRIMER 4F    ---------- ---------- ---------- ---------- ----------
PRIMER 4R    ---------- ---------- ---------- ---------- ----------
```

```
PRIMER 5F    ---------- ---------- ---------- ---------- ----------
PRIMER 5R    ---------- ---------- ---------- ---------- ----------
PRIMER 6F    ---------- ---------- ---------- ---------- ----------
PRIMER 6R    ---------- ---------- ---------- ---------- ----------
PRIMER 7R    ---------- ---------- ---------- ---------- ----------
PRIMER 8F    ---------- ---------- ---------- ---------- ----------

                      860        870        880        890        900
             ....|....| ....|....| ....|....| ....|....| ....|....|
Wuhan-Hu-1   CCATTACAGA TGCTGTAGAC TGTGCACTTG ACCCTCTCTC AGAAACAAAG
B.1.1.7      CCATTACAGA TGCTGTAGAC TGTGCACTTG ACCCTCTCTC AGAAACAAAG
B.1.351      CCATTACAGA TGCTGTAGAC TGTGCACTTG ACCCTCTCTC AGAAACAAAG
P.1          CCATTACAGA TGCTGTAGAC TGTGCACTTG ACCCTCTCTC AGAAACAAAG
B.1.617      CCATTACAGA TGCTGTAGAC TGTGCACTTG ACCCTCTCTC AGAAACAAAG
PRIMER 1F    ---------- ---------- ---------- ---------- ----------
PRIMER 1R    ---------- ---------- ---------- ---------- ---------G
PRIMER 2F    ---------- ---------- ---------- ---------- ----------
PRIMER 2R    ---------- ---------- ---------- ---------- ----------
PRIMER 3F    ---------- ---------- ---------- ---------- ----------
PRIMER 3R    ---------- ---------- ---------- ---------- ----------
PRIMER 4F    ---------- ---------- ---------- ---------- ----------
PRIMER 4R    ---------- ---------- ---------- ---------- ----------
PRIMER 5F    ---------- ---------- ---------- ---------- ----------
PRIMER 5R    ---------- ---------- ---------- ---------- ----------
PRIMER 6F    ---------- ---------- ---------- ---------- ----------
PRIMER 6R    ---------- ---------- ---------- ---------- ----------
PRIMER 7R    ---------- ---------- ---------- ---------- ----------
PRIMER 8F    ---------- ---------- ---------- ---------- ----------

                      910        920        930        940        950
             ....|....| ....|....| ....|....| ....|....| ....|....|
Wuhan-Hu-1   TGTACGTTGA AATCCTTCAC TGTAGAAAAA GGAATCTATC AAACTTCTAA
B.1.1.7      TGTACGTTGA AATCCTTCAC TGTAGAAAAA GGAATCTATC AAACTTCTAA
B.1.351      TGTACGTTGA AATCCTTCAC TGTAGAAAAA GGAATCTATC AAACTTCTAA
P.1          TGTACGTTGA AATCCTTCAC TGTAGAAAAA GGAATCTATC AAACTTCTAA
B.1.617      TGTACGTTGA AATCCTTCAC TGTAGAAAAA GGAATCTATC AAACTTCTAA
PRIMER 1F    ---------- ---------- ---------- ---------- ----------
PRIMER 1R    TGTACGTTGA AATCCTTCAC TGT------- ---------- ----------
PRIMER 2F    ---------- ---------- ---------- ---------- ----------
PRIMER 2R    ---------- ---------- ---------- ---------- ----------
PRIMER 3F    ---------- ---------- ---------- ---------- ----------
PRIMER 3R    ---------- ---------- ---------- ---------- ----------
PRIMER 4F    ---------- ---------- ---------- ---------- ----------
PRIMER 4R    ---------- ---------- ---------- ---------- ----------
PRIMER 5F    ---------- ---------- ---------- ---------- ----------
PRIMER 5R    ---------- ---------- ---------- ---------- ----------
PRIMER 6F    ---------- ---------- ---------- ---------- ----------
PRIMER 6R    ---------- ---------- ---------- ---------- ----------
PRIMER 7R    ---------- ---------- ---------- ---------- ----------
PRIMER 8F    ---------- ---------- ---------- ---------- ----------

                      960        970        980        990       1000
             ....|....| ....|....| ....|....| ....|....| ....|....|
Wuhan-Hu-1   CTTTAGAGTC CAACCAACAG AATCTATTGT TAGATTTCCT AATATTACAA
B.1.1.7      CTTTAGAGTC CAACCAACAG AATCTATTGT TAGATTTCCT AATATTACAA
B.1.351      CTTTAGAGTC CAACCAACAG AATCTATTGT TAGATTTCCT AATATTACAA
P.1          CTTTAGAGTC CAACCAACAG AATCTATTGT TAGATTTCCT AATATTACAA
B.1.617      CTTTAGAGTC CAACCAACAG AATCTATTGT TAGATTTCCT AATATTACAA
PRIMER 1F    ---------- ---------- ---------- ---------- ----------
PRIMER 1R    ---------- ---------- ---------- ---------- ----------
PRIMER 2F    ---------- ---------- ---------- ---------- ----------
PRIMER 2R    ---------- ---------- ---------- ---------- ----------
```

```
PRIMER 3F     ---------- ---------- ---------- ---------- ----------
PRIMER 3R     ---------- ---------- ---------- ---------- ----------
PRIMER 4F     ---------- ---------- ---------- ---------- ----------
PRIMER 4R     ---------- ---------- ---------- ---------- ----------
PRIMER 5F     ---------- ---------- ---------- ---------- ----------
PRIMER 5R     ---------- ---------- ---------- ---------- ----------
PRIMER 6F     ---------- ---------- ---------- ---------- ----------
PRIMER 6R     ---------- ---------- ---------- ---------- ----------
PRIMER 7R     ---------- ---------- ---------- ---------- ----------
PRIMER 8F     ---------- ---------- ---------- ---------- ----------

                    1010       1020       1030       1040       1050
              ....|....| ....|....| ....|....| ....|....| ....|....|
Wuhan-Hu-1    ACTTGTGCCC TTTTGGTGAA GTTTTTAACG CCACCAGATT TGCATCTGTT
B.1.1.7       ACTTGTGCCC TTTTGGTGAA GTTTTTAACG CCACCAGATT TGCATCTGTT
B.1.351       ACTTGTGCCC TTTTGGTGAA GTTTTTAACG CCACCAGATT TGCATCTGTT
P.1           ACTTGTGCCC TTTTGGTGAA GTTTTTAACG CCACCAGATT TGCATCTGTT
B.1.617       ACTTGTGCCC TTTTGGTGAA GTTTTTAACG CCACCAGATT TGCATCTGTT
PRIMER 1F     ---------- ---------- ---------- ---------- ----------
PRIMER 1R     ---------- ---------- ---------- ---------- ----------
PRIMER 2F     ---------- ---------- ---------- ---------- ----------
PRIMER 2R     ---------- ---------- ---------- ---------- ----------
PRIMER 3F     ---------- ---------- ---------- ---------- ----------
PRIMER 3R     ---------- ---------- ---------- ---------- ----------
PRIMER 4F     ---------- ---------- ---------- ---------- ----------
PRIMER 4R     ---------- ---------- ---------- ---------- ----------
PRIMER 5F     ---------- ---------- ---------- ---------- ----------
PRIMER 5R     ---------- ---------- ---------- ---------- ----------
PRIMER 6F     ---------- ---------- ---------- ---------- ----------
PRIMER 6R     ---------- ---------- ---------- ---------- ----------
PRIMER 7R     ---------- ---------- ---------- ---------- ----------
PRIMER 8F     ---------- ---------- ---------- ---------- ----------

                    1060       1070       1080       1090       1100
              ....|....| ....|....| ....|....| ....|....| ....|....|
Wuhan-Hu-1    TATGCTTGGA ACAGGAAGAG AATCAGCAAC TGTGTTGCTG ATTATTCTGT
B.1.1.7       TATGCTTGGA ACAGGAAGAG AATCAGCAAC TGTGTTGCTG ATTATTCTGT
B.1.351       TATGCTTGGA ACAGGAAGAG AATCAGCAAC TGTGTTGCTG ATTATTCTGT
P.1           TATGCTTGGA ACAGGAAGAG AATCAGCAAC TGTGTTGCTG ATTATTCTGT
B.1.617       TATGCTTGGA ACAGGAAGAG AATCAGCAAC TGTGTTGCTG ATTATTCTGT
PRIMER 1F     ---------- ---------- ---------- ---------- ----------
PRIMER 1R     ---------- ---------- ---------- ---------- ----------
PRIMER 2F     ---------- ---------- ---------- ---------- ----------
PRIMER 2R     ---------- ---------- ---------- ---------- ----------
PRIMER 3F     ---------- ---------- ---------- ---------- ----------
PRIMER 3R     ---------- ---------- ---------- ---------- ----------
PRIMER 4F     ---------- ---------- ---------- ---------- ----------
PRIMER 4R     ---------- ---------- ---------- ---------- ----------
PRIMER 5F     ---------- ---------- ---------- ---------- ----------
PRIMER 5R     ---------- ---------- ---------- ---------- ----------
PRIMER 6F     ---------- ---------- ---------- ---------- ----------
PRIMER 6R     ---------- ---------- ---------- ---------- ----------
PRIMER 7R     ---------- ---------- ---------- ---------- ----------
PRIMER 8F     ---------- ---------- ---------- ---------- ----------

                    1110       1120       1130       1140       1150
              ....|....| ....|....| ....|....| ....|....| ....|....|
Wuhan-Hu-1    CCTATATAAT TCCGCATCAT TTTCCACTTT TAAGTGTTAT GGAGTGTCTC
B.1.1.7       CCTATATAAT TCCGCATCAT TTTCCACTTT TAAGTGTTAT GGAGTGTCTC
B.1.351       CCTATATAAT TCTGCATCAT TTTCCACTTT TAAGTGTTAT GGAGTGTCTC
P.1           CCTATATAAT TCCGCATCAT TTTCCACTTT TAAGTGTTAT GGAGTGTCTC
B.1.617       CCTATATAAT TCCGCATCAT TTTCCACTTT TAAGTGTTAT GGAGTGTCTC
```

```
PRIMER 1F     ---------- ---------- ---------- ---------- ----------
PRIMER 1R     ---------- ---------- ---------- ---------- ----------
PRIMER 2F     ---------- ---------- ---------- ---------- ----------
PRIMER 2R     ---------- ---------- ---------- ---------- ----------
PRIMER 3F     ---------- ---------- ---------- ---------- ----------
PRIMER 3R     ---------- ---------- ---------- ---------- ----------
PRIMER 4F     ---------- ---------- ---------- ---------- ----------
PRIMER 4R     ---------- ---------- ---------- ---------- ----------
PRIMER 5F     ---------- ---------- ---------- ---------- ----------
PRIMER 5R     ---------- ---------- ---------- ---------- ----------
PRIMER 6F     ---------- ---------- ---------- ---------- ----------
PRIMER 6R     ---------- ---------- ---------- ---------- ----------
PRIMER 7R     ---------- ---------- ---------- ---------- ----------
PRIMER 8F     ---------- ---------- ---------- ---------- ----------

                       1160       1170       1180       1190       1200
              ....|....| ....|....| ....|....| ....|....| ....|....|
Wuhan-Hu-1    CTACTAAATT AAATGATCTC TGCTTTACTA ATGTCTATGC AGATTCATTT
B.1.1.7       CTACTAAATT AAATGATCTC TGCTTTACTA ATGTCTATGC AGATTCATTT
B.1.351       CTACTAAATT AAATGATCTC TGCTTTACTA ATGTCTATGC AGATTCATTT
P.1           CTACTAAATT AAATGATCTC TGCTTTACTA ATGTCTATGC AGATTCATTT
B.1.617       CTACTAAATT AAATGATCTC TGCTTTACTA ATGTCTATGC AGATTCATTT
PRIMER 1F     ---------- ---------- ---------- ---------- ----------
PRIMER 1R     ---------- ---------- ---------- ---------- ----------
PRIMER 2F     ---------- ---------- ---------- ---------- ----------
PRIMER 2R     ---------- ---------- ---------- ---------- ----------
PRIMER 3F     ---------- ---------- ---------- ---------- ----------
PRIMER 3R     ---------- ---------- ---------- ---------- ----------
PRIMER 4F     ---------- ---------- ---------- ---------- ----------
PRIMER 4R     ---------- ---------- ---------- ---------- ----------
PRIMER 5F     ---------- ---------- ---------- ---------- ----------
PRIMER 5R     ---------- ---------- ---------- ---------- ----------
PRIMER 6F     ---------- ---------- ---------- ---------- ----------
PRIMER 6R     ---------- ---------- ---------- ---------- ----------
PRIMER 7R     ---------- ---------- ---------- ---------- ----------
PRIMER 8F     ---------- ---------- ---------- ---------- ----------

                       1210       1220       1230       1240       1250
              ....|....| ....|....| ....|....| ....|....| ....|....|
Wuhan-Hu-1    GTAATTAGAG GTGATGAAGT CAGACAAATC GCTCCAGGGC AAACTGGAAA
B.1.1.7       GTAATTAGAG GTGATGAAGT CAGACAAATC GCTCCAGGGC AAACTGGAAA
B.1.351       GTAATTAGAG GTGATGAAGT CAGACAAATC GCTCCAGGGC AAACTGGAAA
P.1           GTAATTAGAG GTGATGAAGT CAGACAAATC GCTCCAGGGC AAACTGGAAC
B.1.617       GTAATTAGAG GTGATGAAGT CAGACAAATC GCTCCAGGGC AAACTGGAAA
PRIMER 1F     ---------- ---------- ---------- ---------- ----------
PRIMER 1R     ---------- ---------- ---------- ---------- ----------
PRIMER 2F     ---------- ---------- ---------- ---------- ----------
PRIMER 2R     ---------- ---------- ---------- -------GGC AAACTGGAAA
PRIMER 3F     GTAATTAGAG GTGATGAAGT CAGA------ ---------- ----------
PRIMER 3R     ---------- ---------- ---------- ---------- ----------
PRIMER 4F     ---------- ---------- ---------- ---------- ----------
PRIMER 4R     ---------- ---------- ---------- ---------- ----------
PRIMER 5F     ---------- ---------- ---------- ---------- ----------
PRIMER 5R     ---------- ---------- ---------- ---------- ----------
PRIMER 6F     ---------- ---------- ---------- ---------- ----------
PRIMER 6R     ---------- ---------- ---------- ---------- ----------
PRIMER 7R     ---------- ---------- ---------- ---------- ----------
PRIMER 8F     ---------- ---------- ---------- ---------- ----------

                       1260       1270       1280       1290       1300
              ....|....| ....|....| ....|....| ....|....| ....|....|
Wuhan-Hu-1    GATTGCTGAT TATAATTATA AATTACCAGA TGATTTTACA GGCTGCGTTA
```

```
B.1.1.7      GATTGCTGAT TATAATTATA AATTACCAGA TGATTTTACA GGCTGCGTTA
B.1.351      GATTGCTGAT TATAATTATA AATTACCAGA TGATTTTACA GGCTGCGTTA
P.1          GATTGCTGAT TATAATTATA AATTACCAGA TGATTTTACA GGCTGCGTTA
B.1.617      GATTGCTGAT TATAATTATA AATTACCAGA TGATTTTACA GGCTGCGTTA
PRIMER 1F    ---------- ---------- ---------- ---------- ----------
PRIMER 1R    ---------- ---------- ---------- ---------- ----------
PRIMER 2F    ---------- ---------- ---------- ---------- ----------
PRIMER 2R    GATTGCTGA- ---------- ---------- ---------- ----------
PRIMER 3F    ---------- ---------- ---------- ---------- ----------
PRIMER 3R    ---------- ---------- ---------- ---------- ----------
PRIMER 4F    ---------- ---------- ---------- ---------- ----------
PRIMER 4R    ---------- ---------- ---------- ---------- ----------
PRIMER 5F    ---------- ---------- ---------- ---------- ----------
PRIMER 5R    ---------- ---------- ---------- ---------- ----------
PRIMER 6F    ---------- ---------- ---------- ---------- ----------
PRIMER 6R    ---------- ---------- ---------- ---------- ----------
PRIMER 7R    ---------- ---------- ---------- ---------- ----------
PRIMER 8F    ---------- ---------- ---------- ---------- ----------

                   1310       1320       1330       1340       1350
             ....|....| ....|....| ....|....| ....|....| ....|....|
Wuhan-Hu-1   TAGCTTGGAA TTCTAACAAT CTTGATTCTA AGGTTGGTGG TAATTATAAT
B.1.1.7      TAGCTTGGAA TTCTAACAAT CTTGATTCTA AGGTTGGTGG TAATTATAAT
B.1.351      TAGCTTGGAA TTCTAACAAT CTTGATTCTA AGGTTGGTGG TAATTATAAT
P.1          TAGCTTGGAA TTCTAACAAT CTTGATTCTA AGGTTGGTGG TAATTATAAT
B.1.617      TAGCTTGGAA TTCTAACAAT CTTGATTCTA AGGTTGGTGG TAATTATAAT
PRIMER 1F    ---------- ---------- ---------- ---------- ----------
PRIMER 1R    ---------- ---------- ---------- ---------- ----------
PRIMER 2F    ---------- ---------- ---------- ---------- ----------
PRIMER 2R    ---------- ---------- ---------- ---------- ----------
PRIMER 3F    ---------- ---------- ---------- ---------- ----------
PRIMER 3R    ---------- ---------- ---------- ---------- ----------
PRIMER 4F    ---------- ---------- ---------- ---------- ----------
PRIMER 4R    ---------- ---------- ---------- ---------- ----------
PRIMER 5F    ---------- ---------- ---------- ---------- ----------
PRIMER 5R    ---------- ---------- ---------- ---------- ----------
PRIMER 6F    ---------- ---------- ---------- ---------- ----------
PRIMER 6R    ---------- ---------- ---------- ---------- ----------
PRIMER 7R    ---------- ---------- ---------- ---------- ----------
PRIMER 8F    ---------- ---------- ---------- ---------- ----------

                   1360       1370       1380       1390       1400
             ....|....| ....|....| ....|....| ....|....| ....|....|
Wuhan-Hu-1   TACCTGTATA GATTGTTTAG GAAGTCTAAT CTCAAACCTT TTGAGAGAGA
B.1.1.7      TACCTGTATA GATTGTTTAG GAAGTCTAAT CTCAAACCTT TTGAGAGAGA
B.1.351      TACCTGTATA GATTGTTTAG GAAGTCTAAT CTCAAACCTT TTGAGAGAGA
P.1          TACCTGTATA GATTGTTTAG GAAGTCTAAT CTCAAACCTT TTGAGAGAGA
B.1.617      TACCGGTATA GATTGTTTAG GAAGTCTAAT CTCAAACCTT TTGAGAGAGA
PRIMER 1F    ---------- ---------- ---------- ---------- ----------
PRIMER 1R    ---------- ---------- ---------- ---------- ----------
PRIMER 2F    ---------- ---------- ---------- ---------- ----------
PRIMER 2R    ---------- ---------- ---------- ---------- ----------
PRIMER 3F    ---------- ---------- ---------- ---------- ----------
PRIMER 3R    ---------- ---------- ---------- ---------- ----------
PRIMER 4F    ---------- ---------- ---------- ---------- ----------
PRIMER 4R    ---------- ---------- ---------- ---------- ----------
PRIMER 5F    ---------- ---------- ---------- ---------- ----------
PRIMER 5R    ---------- ---------- ---------- ---------- ----------
PRIMER 6F    ---------- ---------- ---------- ---------- ----------
PRIMER 6R    ---------- ---------- ---------- ---------- ----------
PRIMER 7R    ---------- ---------- ---------- ---------- ----------
PRIMER 8F    ---------- ---------- ---------- ---------- ----------
```

```
                   1410       1420       1430       1440       1450
             ....|....| ....|....| ....|....| ....|....| ....|....|
Wuhan-Hu-1   TATTTCAACT GAAATCTATC AGGCCGGTAG CACACCTTGT AATGGTGTTG
B.1.1.7      TATTTCAACT GAAATCTATC AGGCCGGTAG CACACCTTGT AATGGTGTTG
B.1.351      TATTTCAACT GAAATCTATC AGGCCGGTAG CACACCTTGT AATGGTGTTA
P.1          TATTTCAACT GAAATCTATC AGGCCGGTAG CACACCTTGT AATGGTGTTA
B.1.617      TATTTCAACT GAAATCTATC AGGCCGGTAG CAAACCTTGT AATGGTGTTG
PRIMER 1F    ---------- ---------- ---------- ---------- ----------
PRIMER 1R    ---------- ---------- ---------- ---------- ----------
PRIMER 2F    ---------- ---------- ---------- ---------- ----------
PRIMER 2R    ---------- ---------- ---------- ---------- ----------
PRIMER 3F    ---------- ---------- ---------- ---------- ----------
PRIMER 3R    ---------- ---------- ---------- ---------- ----------
PRIMER 4F    ---------- ---------- ---------- ---------- ----------
PRIMER 4R    ---------- ---------- ---------- ---------- ----------
PRIMER 5F    ---------- ---------- ---------- ---------- ----------
PRIMER 5R    ---------- ---------- ---------- ---------- ----------
PRIMER 6F    ---------- ---------- ---------- ---------- ----------
PRIMER 6R    ---------- ---------- ---------- ---------- ----------
PRIMER 7R    ---------- ---------- ---------- ---------- ----------
PRIMER 8F    ---------- ---------- ---------- ---------- ----------

                   1460       1470       1480       1490       1500
             ....|....| ....|....| ....|....| ....|....| ....|....|
Wuhan-Hu-1   AAGGTTTTAA TTGTTACTTT CCTTTACAAT CATATGGTTT CCAACCCACT
B.1.1.7      AAGGTTTTAA TTGTTACTTT CCTTTACAAT CATATGGTTT CCAACCCACT
B.1.351      AAGGTTTTAA TTGTTACTTT CCTTTACAAT CATATGGTTT CCAACCCACT
P.1          AAGGTTTTAA TTGTTACTTT CCTTTACAAT CATATGGTTT CCAACCCACT
B.1.617      AAGGTTTTAA TTGTTACTTT CCTTTACAAT CATATGGTTT CCAACCCACT
PRIMER 1F    ---------- ---------- ---------- ---------- ----------
PRIMER 1R    ---------- ---------- ---------- ---------- ----------
PRIMER 2F    ---------- ---------- ---------- ---------- ----------
PRIMER 2R    ---------- ---------- ---------- ---------- ----------
PRIMER 3F    ---------- ---------- ---------- ---------- ----------
PRIMER 3R    ---------- ---------- ---------- ---------- ----------
PRIMER 4F    ---------- ---------- ---------- ---------- ----------
PRIMER 4R    ---------- ---------- ---------- ---------- ----------
PRIMER 5F    ---------- ---------- ---------- ---------- ----------
PRIMER 5R    ---------- ---------- ---------- ---------- ----------
PRIMER 6F    ---------- ---------- ---------- ---------- ----------
PRIMER 6R    ---------- ---------- ---------- ---------- ----------
PRIMER 7R    ---------- ---------- ---------- ---------- ----------
PRIMER 8F    ---------- ---------- ---------- ---------- ----------

                   1510       1520       1530       1540       1550
             ....|....| ....|....| ....|....| ....|....| ....|....|
Wuhan-Hu-1   AATGGTGTTG GTTACCAACC ATACAGAGTA GTAGTACTTT CTTTTGAACT
B.1.1.7      TATGGTGTTG GTTACCAACC ATACAGAGTA GTAGTACTTT CTTTTGAACT
B.1.351      TATGGTGTTG GTTACCAACC ATACAGAGTA GTAGTACTTT CTTTTGAACT
P.1          TATGGTGTTG GTTACCAACC ATACAGAGTA GTAGTACTTT CTTTTGAACT
B.1.617      AATGGTGTTG GTTACCAACC ATACAGAGTA GTAGTACTTT CTTTTGAACT
PRIMER 1F    ---------- ---------- ---------- ---------- ----------
PRIMER 1R    ---------- ---------- ---------- ---------- ----------
PRIMER 2F    ---------- ---------- ---------- ---------- ----------
PRIMER 2R    ---------- ---------- ---------- ---------- ----------
PRIMER 3F    ---------- ---------- ---------- ---------- ----------
PRIMER 3R    ---------- ---------- ---------- ---------- ----------
PRIMER 4F    ---------- ---------- ---------- ---------- ----------
PRIMER 4R    ---------- ---------- ---------- ---------- ----------
PRIMER 5F    ---------- ---------- ---------- ---------- ----------
PRIMER 5R    ---------- ---------- ---------- ---------- ----------
```

```
PRIMER 6F    ---------- ---------- ---------- ---------- ----------
PRIMER 6R    ---------- ---------- ---------- ---------- ----------
PRIMER 7R    ---------- ---------- ---------- ---------- ----------
PRIMER 8F    ---------- ---------- ---------- ---------- ----------

                     1560       1570       1580       1590       1600
             ....|....| ....|....| ....|....| ....|....| ....|....|
Wuhan-Hu-1   TCTACATGCA CCAGCAACTG TTTGTGGACC TAAAAAGTCT ACTAATTTGG
B.1.1.7      TCTACATGCA CCAGCAACTG TTTGTGGACC TAAAAAGTCT ACTAATTTGG
B.1.351      TCTACATGCA CCAGCAACTG TTTGTGGACC TAAAAAGTCT ACTAATTTGG
P.1          TCTACATGCA CCAGCAACTG TTTGTGGACC TAAAAAGTCT ACTAATTTGG
B.1.617      TCTACATGCA CCAGCAACTG TTTGTGGACC TAAAAAGTCT ACTAATTTGG
PRIMER 1F    ---------- ---------- ---------- ---------- ----------
PRIMER 1R    ---------- ---------- ---------- ---------- ----------
PRIMER 2F    ---------- ---------- ---------- ---------- ----------
PRIMER 2R    ---------- ---------- ---------- ---------- ----------
PRIMER 3F    ---------- ---------- ---------- ---------- ----------
PRIMER 3R    ---------- ---------- ---------- ---------- ----------
PRIMER 4F    ---------- ---------- ---------- ---------- ----------
PRIMER 4R    ---------- ---------- ---------- ---------- ----------
PRIMER 5F    ---------- ---------- ---------- ---------- ----------
PRIMER 5R    ---------- ---------- ---------- ---------- ----------
PRIMER 6F    ---------- ---------- ---------- ---------- ----------
PRIMER 6R    ---------- ---------- ---------- ---------- ----------
PRIMER 7R    ---------- ---------- ---------- ---------- ----------
PRIMER 8F    ---------- ---------- ---------- ---------- ----------

                     1610       1620       1630       1640       1650
             ....|....| ....|....| ....|....| ....|....| ....|....|
Wuhan-Hu-1   TTAAAAACAA ATGTGTCAAT TTCAACTTCA ATGGTTTAAC AGGCACAGGT
B.1.1.7      TTAAAAACAA ATGTGTCAAT TTCAACTTCA ATGGTTTAAC AGGCACAGGT
B.1.351      TTAAAAACAA ATGTGTCAAT TTCAACTTCA ATGGTTTAAC AGGCACAGGT
P.1          TTAAAAACAA ATGTGTCAAT TTCAACTTCA ATGGTTTAAC AGGCACAGGT
B.1.617      TTAAAAACAA ATGTGTCAAT TTCAACTTCA ATGGTTTAAC AGGCACAGGT
PRIMER 1F    ---------- ---------- ---------- ---------- ----------
PRIMER 1R    ---------- ---------- ---------- ---------- ----------
PRIMER 2F    ---------- ---------- ---------- ---------- ----------
PRIMER 2R    ---------- ---------- ---------- ---------- ----------
PRIMER 3F    ---------- ---------- ---------- ---------- ----------
PRIMER 3R    ---------- ---------- ---------- ---------- ----------
PRIMER 4F    ---------- ---------- ---------- ---------- ----------
PRIMER 4R    ---------- ---------- ---------- ---------- ----------
PRIMER 5F    ---------- ---------- ---------- ---------- ----------
PRIMER 5R    ---------- ---------- ---------- ---------- ----------
PRIMER 6F    ---------- ---------- ---------- ---------- ----------
PRIMER 6R    ---------- ---------- ---------- ---------- ----------
PRIMER 7R    ---------- ---------- ---------- ---------- ----------
PRIMER 8F    ---------- ---------- ---------- ---------- ----------

                     1660       1670       1680       1690       1700
             ....|....| ....|....| ....|....| ....|....| ....|....|
Wuhan-Hu-1   GTTCTTACTG AGTCTAACAA AAAGTTTCTG CCTTTCCAAC AATTTGGCAG
B.1.1.7      GTTCTTACTG AGTCTAACAA AAAGTTTCTG CCTTTCCAAC AATTTGGCAG
B.1.351      GTTCTTACTG AGTCTAACAA AAAGTTTCTG CCTTTCCAAC AATTTGGCAG
P.1          GTTCTTACTG AGTCTAACAA AAAGTTTCTG CCTTTCCAAC AATTTGGCAG
B.1.617      GTTCTTACTG AGTCTAACAA AAAGTTTCTG CCTTTCCAAC AATTTGGCAG
PRIMER 1F    ---------- ---------- ---------- ---------- ----------
PRIMER 1R    ---------- ---------- ---------- ---------- ----------
PRIMER 2F    ---------- ---------- ---------- ---------- ----------
PRIMER 2R    ---------- ---------- ---------- ---------- ----------
PRIMER 3F    ---------- ---------- ---------- ---------- ----------
PRIMER 3R    ---------- ---------- ---------- ---------- ----------
```

```
PRIMER 4F    ---------- ---------- ---------- ---------- ----------
PRIMER 4R    ---------- ---------- ---------- ---------- ----------
PRIMER 5F    ---------- ---------- ---------- ---------- ----------
PRIMER 5R    ---------- ---------- ---------- ---------- ----------
PRIMER 6F    ---------- ---------- ---------- ---------- ----------
PRIMER 6R    ---------- ---------- ---------- ---------- ----------
PRIMER 7R    ---------- ---------- ---------- ---------- ----------
PRIMER 8F    ---------- ---------- ---------- ---------- ----------

                     1710       1720       1730       1740       1750
             ....|....| ....|....| ....|....| ....|....| ....|....|
Wuhan-Hu-1   AGACATTGCT GACACTACTG ATGCTGTCCG TGATCCACAG ACACTTGAGA
B.1.1.7      AGACATTGAT GACACTACTG ATGCTGTCCG TGATCCACAG ACACTTGAGA
B.1.351      AGACATTGCT GACACTACTG ATGCTGTCCG TGATCCACAG ACACTTGAGA
P.1          AGACATTGCT GACACTACTG ATGCTGTCCG TGATCCACAG ACACTTGAGA
B.1.617      AGACATTGCT GACACTACTG ATGCTGTCCG TGATCCACAG ACACTTGAGA
PRIMER 1F    ---------- ---------- ---------- ---------- ----------
PRIMER 1R    ---------- ---------- ---------- ---------- ----------
PRIMER 2F    ---------- ---------- ---------- ---------- ----------
PRIMER 2R    ---------- ---------- ---------- ---------- ----------
PRIMER 3F    ---------- ---------- ---------- ---------- ----------
PRIMER 3R    ---------- ---------- ---------- ---------- ----------
PRIMER 4F    ---------- ---------- ---------- ---------- ----------
PRIMER 4R    ---------- ---------- ---------- ---------- ----------
PRIMER 5F    ---------- ---------- ---------- ---------- ----------
PRIMER 5R    ---------- ---------- ---------- ---------- ----------
PRIMER 6F    ---------- ---------- ---------- ---------- ----------
PRIMER 6R    ---------- ---------- ---------- ---------- ----------
PRIMER 7R    ---------- ---------- ---------- ---------- ----------
PRIMER 8F    ---------- ---------- ---------- ---------- ----------

                     1760       1770       1780       1790       1800
             ....|....| ....|....| ....|....| ....|....| ....|....|
Wuhan-Hu-1   TTCTTGACAT TACACCATGT TCTTTTGGTG GTGTCAGTGT TATAACACCA
B.1.1.7      TTCTTGACAT TACACCATGT TCTTTTGGTG GTGTCAGTGT TATAACACCA
B.1.351      TTCTTGACAT TACACCATGT TCTTTTGGTG GTGTCAGTGT TATAACACCA
P.1          TTCTTGACAT TACACCATGT TCTTTTGGTG GTGTCAGTGT TATAACACCA
B.1.617      TTCTTGACAT TACACCATGT TCTTTTGGTG GTGTCAGTGT TATAACACCA
PRIMER 1F    ---------- ---------- ---------- ---------- ----------
PRIMER 1R    ---------- ---------- ---------- ---------- ----------
PRIMER 2F    ---------- ---------- ---------- ---------- ----------
PRIMER 2R    ---------- ---------- ---------- ---------- ----------
PRIMER 3F    ---------- ---------- ---------- ---------- ----------
PRIMER 3R    ---------- ---------- ---------- ---------- ----------
PRIMER 4F    ---------- ---------- ---------- ---------- ----------
PRIMER 4R    ---------- ---------- ---------- ---------- ----------
PRIMER 5F    ---------- ---------- ---------- ---------- ----------
PRIMER 5R    ---------- ---------- ---------- ---------- ----------
PRIMER 6F    ---------- ---------- ---------- ---------- ----------
PRIMER 6R    ---------- ---------- ---------- ---------- ----------
PRIMER 7R    ---------- ---------- ---------- ---------- ----------
PRIMER 8F    ---------- ---------- ---------- ---------- ----------

                     1810       1820       1830       1840       1850
             ....|....| ....|....| ....|....| ....|....| ....|....|
Wuhan-Hu-1   GGAACAAATA CTTCTAACCA GGTTGCTGTT CTTTATCAGG ATGTTAACTG
B.1.1.7      GGAACAAATA CTTCTAACCA GGTTGCTGTT CTTTATCAGG GTGTTAACTG
B.1.351      GGAACAAATA CTTCTAACCA GGTTGCTGTT CTTTATCAGG GTGTTAACTG
P.1          GGAACAAATA CTTCTAACCA GGTTGCTGTT CTTTATCAGG GTGTTAACTG
B.1.617      GGAACAAATA CTTCTAACCA GGTTGCTGTT CTTTATCAGG GTGTTAACTG
PRIMER 1F    ---------- ---------- ---------- ---------- ----------
PRIMER 1R    ---------- ---------- ---------- ---------- ----------
```

```
PRIMER 2F    ---------- ---------- ---------- ---------- ----------
PRIMER 2R    ---------- ---------- ---------- ---------- ----------
PRIMER 3F    ---------- ---------- ---------- ---------- ----------
PRIMER 3R    ---------- ---------- ---------- ---------- ----------
PRIMER 4F    ---------- ---------- ---------- ---------- ----------
PRIMER 4R    ---------- ---------- ---------- ---------- ----------
PRIMER 5F    ---------- ---------- ---------- ---------- ----------
PRIMER 5R    ---------- ---------- ---------- ---------- ----------
PRIMER 6F    ---------- ---------- ---------- ---------- ----------
PRIMER 6R    ---------- ---------- ---------- ---------- ----------
PRIMER 7R    ---------- ---------- ---------- ---------- ----------
PRIMER 8F    ---------- ---------- ---------- ---------- ----------

                  1860       1870       1880       1890       1900
             ....|....| ....|....| ....|....| ....|....| ....|....|
Wuhan-Hu-1   CACAGAAGTC CCTGTTGCTA TTCATGCAGA TCAACTTACT CCTACTTGGC
B.1.1.7      CACAGAAGTC CCTGTTGCTA TTCATGCAGA TCAACTTACT CCTACTTGGC
B.1.351      CACAGAAGTC CCTGTTGCTA TTCATGCAGA TCAACTTACT CCTACTTGGC
P.1          CACAGAAGTC CCTGTTGCTA TTCATGCAGA TCAACTTACT CCTACTTGGC
B.1.617      CACAGAAGTC CCTGTTGCTA TTCATGCAGA TCAACTTACT CCTACTTGGC
PRIMER 1F    ---------- ---------- ---------- ---------- ----------
PRIMER 1R    ---------- ---------- ---------- ---------- ----------
PRIMER 2F    ---------- ---------- ---------- ---------- ----------
PRIMER 2R    ---------- ---------- ---------- ---------- ----------
PRIMER 3F    ---------- ---------- ---------- ---------- ----------
PRIMER 3R    ---------- ---------- ---------- ---------- ----------
PRIMER 4F    ---------- ---------- ---------- ---------- ----CTTGGC
PRIMER 4R    ---------- ---------- ---------- ---------- ----------
PRIMER 5F    ---------- ---------- ---------- ---------- ----------
PRIMER 5R    ---------- ---------- ---------- ---------- ----------
PRIMER 6F    ---------- ---------- ---------- ---------- ----------
PRIMER 6R    ---------- ---------- ---------- ---------- ----------
PRIMER 7R    ---------- ---------- ---------- ---------- ----------
PRIMER 8F    ---------- ---------- ---------- ---------- ----------

                  1910       1920       1930       1940       1950
             ....|....| ....|....| ....|....| ....|....| ....|....|
Wuhan-Hu-1   GTGTTTATTC TACAGGTTCT AATGTTTTTC AAACACGTGC AGGCTGTTTA
B.1.1.7      GTGTTTATTC TACAGGTTCT AATGTTTTTC AAACACGTGC AGGCTGTTTA
B.1.351      GTGTTTATTC TACAGGTTCT AATGTTTTTC AAACACGTGC AGGCTGTTTA
P.1          GTGTTTATTC TACAGGTTCT AATGTTTTTC AAACACGTGC AGGCTGTTTA
B.1.617      GTGTTTATTC TACAGGTTCT AATGTTTTTC AAACACGTGC AGGCTGTTTA
PRIMER 1F    ---------- ---------- ---------- ---------- ----------
PRIMER 1R    ---------- ---------- ---------- ---------- ----------
PRIMER 2F    ---------- ---------- ---------- ---------- ----------
PRIMER 2R    ---------- ---------- ---------- ---------- ----------
PRIMER 3F    ---------- ---------- ---------- ---------- ----------
PRIMER 3R    ---------- ---------- ---------- ---------- ----------
PRIMER 4F    GTGTTTATTC TACAG----- ---------- ---------- ----------
PRIMER 4R    ---------- ---------- ---------- ---------- ----------
PRIMER 5F    ---------- ---------- ---------- ---------- ----------
PRIMER 5R    ---------- ---------- ---------- ---------- ----------
PRIMER 6F    ---------- ---------- ---------- ---------- ----------
PRIMER 6R    ---------- ---------- ---------- ---------- ----------
PRIMER 7R    ---------- ---------- ---------- ---------- ----------
PRIMER 8F    ---------- ---------- ---------- ---------- ----------

                  1960       1970       1980       1990       2000
             ....|....| ....|....| ....|....| ....|....| ....|....|
Wuhan-Hu-1   ATAGGGGCTG AACATGTCAA CAACTCATAT GAGTGTGACA TACCCATTGG
B.1.1.7      ATAGGGGCTG AACATGTCAA CAACTCATAT GAGTGTGACA TACCCATTGG
B.1.351      ATAGGGGCTG AACATGTCAA CAACTCATAT GAGTGTGACA TACCCATTGG
```

```
P.1          ATAGGGGCTG AATATGTCAA CAACTCATAT GAGTGTGACA TACCCATTGG
B.1.617      ATAGGGGCTG AACATGTCAA CAACTCATAT GAGTGTGACA TACCCATTGG
PRIMER 1F    ---------- ---------- ---------- ---------- ----------
PRIMER 1R    ---------- ---------- ---------- ---------- ----------
PRIMER 2F    ---------- ---------- ---------- ---------- ----------
PRIMER 2R    ---------- ---------- ---------- ---------- ----------
PRIMER 3F    ---------- ---------- ---------- ---------- ----------
PRIMER 3R    ---------- ---------- ---------- ---------- ----------
PRIMER 4F    ---------- ---------- ---------- ---------- ----------
PRIMER 4R    ---------- ---------- ---------- ---------- ----------
PRIMER 5F    ---------- ---------- ---------- ---------- ----------
PRIMER 5R    ---------- ---------- ---------- ---------- ----------
PRIMER 6F    ---------- ---------- ---------- ---------- ----------
PRIMER 6R    ---------- ---------- ---------- ---------- ----------
PRIMER 7R    ---------- ---------- ---------- ---------- ----------
PRIMER 8F    ---------- ---------- ---------- ---------- ----------

                     2010       2020       2030       2040       2050
             ....|....| ....|....| ....|....| ....|....| ....|....|
Wuhan-Hu-1   TGCAGGTATA TGCGCTAGTT ATCAGACTCA GACTAATTCT CCTCGGCGGG
B.1.1.7      TGCAGGTATA TGCGCTAGTT ATCAGACTCA GACTAATTCT CATCGGCGGG
B.1.351      TGCAGGTATA TGCGCTAGTT ATCAGACTCA GACTAATTCT CCTCGGCGGG
P.1          TGCAGGTATA TGCGCTAGTT ATCAGACTCA GACTAATTCT CCTCGGCGGG
B.1.617      TGCAGGTATA TGCGCTAGTT ATCAGACTCA GACTAATTCT CGTCGGCGGG
PRIMER 1F    ---------- ---------- ---------- ---------- ----------
PRIMER 1R    ---------- ---------- ---------- ---------- ----------
PRIMER 2F    ---------- ---------- ---------- ---------- ----------
PRIMER 2R    ---------- ---------- ---------- ---------- ----------
PRIMER 3F    ---------- ---------- ---------- ---------- ----------
PRIMER 3R    ---------- ---------- ---------- ---------- ----------
PRIMER 4F    ---------- ---------- ---------- ---------- ----------
PRIMER 4R    ---------- ---------- ---------- ---------- ----------
PRIMER 5F    ---------- ---------- ---------- ---------- ----------
PRIMER 5R    ---------- ---------- ---------- ---------- ----------
PRIMER 6F    ---------- ---------- ---------- ---------- ----------
PRIMER 6R    ---------- ---------- ---------- ---------- ----------
PRIMER 7R    ---------- ---------- ---------- ---------- ----------
PRIMER 8F    ---------- ---------- ---------- ---------- ----------

                     2060       2070       2080       2090       2100
             ....|....| ....|....| ....|....| ....|....| ....|....|
Wuhan-Hu-1   CACGTAGTGT AGCTAGTCAA TCCATCATTG CCTACACTAT GTCACTTGGT
B.1.1.7      CACGTAGTGT AGCTAGTCAA TCCATCATTG CCTACACTAT GTCACTTGGT
B.1.351      CACGTAGTGT AGCTAGTCAA TCCATCATTG CCTACACTAT GTCACTTGGT
P.1          CACGTAGTGT AGCTAGTCAA TCCATCATTG CCTACACTAT GTCACTTGGT
B.1.617      CACGTAGTGT AGCTAGTCAA TCCATCATTG CCTACACTAT GTCACTTGGT
PRIMER 1F    ---------- ---------- ---------- ---------- ----------
PRIMER 1R    ---------- ---------- ---------- ---------- ----------
PRIMER 2F    ---------- ---------- ---------- ---------- ----------
PRIMER 2R    ---------- ---------- ---------- ---------- ----------
PRIMER 3F    ---------- ---------- ---------- ---------- ----------
PRIMER 3R    ---------- ---------- TCCATCATTG CCTACACTAT GT--------
PRIMER 4F    ---------- ---------- ---------- ---------- ----------
PRIMER 4R    ---------- ---------- ---------- ---------- ----------
PRIMER 5F    ---------- ---------- ---------- ---------- ----------
PRIMER 5R    ---------- ---------- ---------- ---------- ----------
PRIMER 6F    ---------- ---------- ---------- ---------- ----------
PRIMER 6R    ---------- ---------- ---------- ---------- ----------
PRIMER 7R    ---------- ---------- ---------- ---------- ----------
PRIMER 8F    ---------- ---------- ---------- ---------- ----------

                     2110       2120       2130       2140       2150
```

```
              ....|....| ....|....| ....|....| ....|....| ....|....|
Wuhan-Hu-1    GCAGAAAATT CAGTTGCTTA CTCTAATAAC TCTATTGCCA TACCCACAAA
B.1.1.7       GCAGAAAATT CAGTTGCTTA CTCTAATAAC TCTATTGCCA TACCCATAAA
B.1.351       GTAGAAAATT CAGTTGCTTA CTCTAATAAC TCTATTGCCA TACCCACAAA
P.1           GCAGAAAATT CAGTTGCTTA CTCTAATAAC TCTATTGCCA TACCCACAAA
B.1.617       GCAGAAAATT CAGTTGCTTA CTCTAATAAC TCTATTGCCA TACCCACAAA
PRIMER 1F     ---------- ---------- ---------- ---------- ----------
PRIMER 1R     ---------- ---------- ---------- ---------- ----------
PRIMER 2F     ---------- ---------- ---------- ---------- ----------
PRIMER 2R     ---------- ---------- ---------- ---------- ----------
PRIMER 3F     ---------- ---------- ---------- ---------- ----------
PRIMER 3R     ---------- ---------- ---------- ---------- ----------
PRIMER 4F     ---------- ---------- ---------- ---------- ----------
PRIMER 4R     ---------- ---------- ---------- ---------- ----------
PRIMER 5F     ---------- ---------- ---------- ---------- ----------
PRIMER 5R     ---------- ---------- ---------- ---------- ----------
PRIMER 6F     ---------- ---------- ---------- ---------- ----------
PRIMER 6R     ---------- ---------- ---------- ---------- ----------
PRIMER 7R     ---------- ---------- ---------- ---------- ----------
PRIMER 8F     ---------- ---------- ---------- ---------- ----------

                       2160       2170       2180       2190       2200
              ....|....| ....|....| ....|....| ....|....| ....|....|
Wuhan-Hu-1    TTTTACTATT AGTGTTACCA CAGAAATTCT ACCAGTGTCT ATGACCAAGA
B.1.1.7       TTTTACTATT AGTGTTACCA CAGAAATTCT ACCAGTGTCT ATGACCAAGA
B.1.351       TTTTACTATT AGTGTTACCA CAGAAATTCT ACCAGTGTCT ATGACCAAGA
P.1           TTTTACTATT AGTGTTACCA CAGAAATTCT ACCAGTGTCT ATGACCAAGA
B.1.617       TTTTACTATT AGTGTTACCA CAGAAATTCT ACCAGTGTCT ATGACCAAGA
PRIMER 1F     ---------- ---------- ---------- ---------- ----------
PRIMER 1R     ---------- ---------- ---------- ---------- ----------
PRIMER 2F     ---------- ---------- ---------- ---------- ----------
PRIMER 2R     ---------- ---------- ---------- ---------- ----------
PRIMER 3F     ---------- ---------- ---------- ---------- ----------
PRIMER 3R     ---------- ---------- ---------- ---------- ----------
PRIMER 4F     ---------- ---------- ---------- ---------- ----------
PRIMER 4R     ---------- ---------- ---------- ---------- ----------
PRIMER 5F     ---------- ---------- ---------- ---------- ----------
PRIMER 5R     ---------- ---------- ---------- ---------- ----------
PRIMER 6F     ---------- ---------- ---------- ---------- ----------
PRIMER 6R     ---------- ---------- ---------- ---------- ----------
PRIMER 7R     ---------- ---------- ---------- ---------- ----------
PRIMER 8F     ---------- ---------- ---------- ---------- ----------

                       2210       2220       2230       2240       2250
              ....|....| ....|....| ....|....| ....|....| ....|....|
Wuhan-Hu-1    CATCAGTAGA TTGTACAATG TACATTTGTG GTGATTCAAC TGAATGCAGC
B.1.1.7       CATCAGTAGA TTGTACAATG TACATTTGTG GTGATTCAAC TGAATGCAGC
B.1.351       CATCAGTAGA TTGTACAATG TACATTTGTG GTGATTCAAC TGAATGCAGC
P.1           CATCAGTAGA TTGTACAATG TACATTTGTG GTGATTCAAC TGAATGCAGC
B.1.617       CATCAGTAGA TTGTACAATG TACATTTGTG GTGATTCAAC TGAATGCAGC
PRIMER 1F     ---------- ---------- ---------- ---------- ----------
PRIMER 1R     ---------- ---------- ---------- ---------- ----------
PRIMER 2F     ---------- ---------- ---------- ---------- ----------
PRIMER 2R     ---------- ---------- ---------- ---------- ----------
PRIMER 3F     ---------- ---------- ---------- ---------- ----------
PRIMER 3R     ---------- ---------- ---------- ---------- ----------
PRIMER 4F     ---------- ---------- ---------- ---------- ----------
PRIMER 4R     ---------- ---------- ---------- ---------- ----------
PRIMER 5F     ---------- ---------- ---------- ---------- ----------
PRIMER 5R     ---------- ---------- ---------- ---------- ----------
PRIMER 6F     ---------- ---------- ---------- ---------- ----------
PRIMER 6R     ---------- ---------- ---------- ---------- ----------
```

```
PRIMER 7R    ---------- ---------- ---------- ---------- ----------
PRIMER 8F    ---------- ---------- ---------- ---------- ----------
```

```
                     2260       2270       2280       2290       2300
             ....|....| ....|....| ....|....| ....|....| ....|....|
Wuhan-Hu-1   AATCTTTTGT TGCAATATGG CAGTTTTTGT ACACAATTAA ACCGTGCTTT
B.1.1.7      AATCTTTTGT TGCAATATGG CAGTTTTTGT ACACAATTAA ACCGTGCTTT
B.1.351      AATCTTTTGT TGCAATATGG CAGTTTTTGT ACACAATTAA ACCGTGCTTT
P.1          AATCTTTTGT TGCAATATGG CAGTTTTTGT ACACAATTAA ACCGTGCTTT
B.1.617      AATCTTTTGT TGCAATATGG CAGTTTTTGT ACACAATTAA ACCGTGCTTT
PRIMER 1F    ---------- ---------- ---------- ---------- ----------
PRIMER 1R    ---------- ---------- ---------- ---------- ----------
PRIMER 2F    ---------- ---------- ---------- ---------- ----------
PRIMER 2R    ---------- ---------- ---------- ---------- ----------
PRIMER 3F    ---------- ---------- ---------- ---------- ----------
PRIMER 3R    ---------- ---------- ---------- ---------- ----------
PRIMER 4F    ---------- ---------- ---------- ---------- ----------
PRIMER 4R    ---------- ---------- ---------- ---------- ----------
PRIMER 5F    ---------- ---------- ---------- ---------- ----------
PRIMER 5R    ---------- ---------- ---------- ---------- ----------
PRIMER 6F    ---------- ---------- ---------- ---------- ----------
PRIMER 6R    ---------- ---------- ---------- ---------- ----------
PRIMER 7R    ---------- ---------- ---------- ---------- ----------
PRIMER 8F    ---------- ---------- ---------- ---------- ----------
```

```
                     2310       2320       2330       2340       2350
             ....|....| ....|....| ....|....| ....|....| ....|....|
Wuhan-Hu-1   AACTGGAATA GCTGTTGAAC AAGACAAAAA CACCCAAGAA GTTTTTGCAC
B.1.1.7      AACTGGAATA GCTGTTGAAC AAGACAAAAA CACCCAAGAA GTTTTTGCAC
B.1.351      AACTGGAATA GCTGTTGAAC AAGACAAAAA CACCCAAGAA GTTTTTGCAC
P.1          AACTGGAATA GCTGTTGAAC AAGACAAAAA CACCCAAGAA GTTTTTGCAC
B.1.617      AACTGGAATA GCTGTTGAAC AAGACAAAAA CACCCAAGAA GTTTTTGCAC
PRIMER 1F    ---------- ---------- ---------- ---------- ----------
PRIMER 1R    ---------- ---------- ---------- ---------- ----------
PRIMER 2F    ---------- ---------- ---------- ---------- ----------
PRIMER 2R    ---------- ---------- ---------- ---------- ----------
PRIMER 3F    ---------- ---------- ---------- ---------- ----------
PRIMER 3R    ---------- ---------- ---------- ---------- ----------
PRIMER 4F    ---------- ---------- ---------- ---------- ----------
PRIMER 4R    ---------- ---------- ---------- ---------- ----------
PRIMER 5F    ---------- ---------- ---------- ---------- ----------
PRIMER 5R    ---------- ---------- ---------- ---------- ----------
PRIMER 6F    ---------- ---------- ---------- ---------- ----------
PRIMER 6R    ---------- ---------- ---------- ---------- ----------
PRIMER 7R    ---------- ---------- ---------- ---------- ----------
PRIMER 8F    ---------- ---------- ---------- ---------- ----------
```

```
                     2360       2370       2380       2390       2400
             ....|....| ....|....| ....|....| ....|....| ....|....|
Wuhan-Hu-1   AAGTCAAACA AATTTACAAA ACACCACCAA TTAAAGATTT TGGTGGTTTT
B.1.1.7      AAGTCAAACA AATTTACAAA ACACCACCAA TTAAAGATTT TGGTGGTTTT
B.1.351      AAGTCAAACA AATTTACAAA ACACCACCAA TTAAAGATTT TGGTGGTTTT
P.1          AAGTCAAACA AATTTACAAA ACACCACCAA TTAAAGATTT TGGTGGTTTT
B.1.617      AAGTCAAACA AATTTACAAA ACACCACCAA TTAAAGATTT TGGTGGTTTT
PRIMER 1F    ---------- ---------- ---------- ---------- ----------
PRIMER 1R    ---------- ---------- ---------- ---------- ----------
PRIMER 2F    ---------- ---------- ---------- ---------- ----------
PRIMER 2R    ---------- ---------- ---------- ---------- ----------
PRIMER 3F    ---------- ---------- ---------- ---------- ----------
PRIMER 3R    ---------- ---------- ---------- ---------- ----------
PRIMER 4F    ---------- ---------- ---------- ---------- ----------
PRIMER 4R    ---------- ---------- ---------- ---------- ----------
```

```
PRIMER 5F     ---------- ---------- ---------- ---------- ----------
PRIMER 5R     ---------- ---------- ---------- ---------- ----------
PRIMER 6F     ---------- ---------- ---------- ---------- ----------
PRIMER 6R     ---------- ---------- ---------- ---------- ----------
PRIMER 7R     ---------- ---------- ---------- ---------- ----------
PRIMER 8F     ---------- ---------- ---------- ---------- ----------

                    2410       2420       2430       2440       2450
              ....|....| ....|....| ....|....| ....|....| ....|....|
Wuhan-Hu-1    AATTTTTCAC AAATATTACC AGATCCATCA AAACCAAGCA AGAGGTCATT
B.1.1.7       AATTTTTCAC AAATATTACC AGATCCATCA AAACCAAGCA AGAGGTCATT
B.1.351       AATTTTTCAC AAATATTACC AGATCCATCA AAACCAAGCA AGAGGTCATT
P.1           AATTTTTCAC AAATATTACC AGATCCATCA AAACCAAGCA AGAGGTCATT
B.1.617       AATTTTTCAC AAATATTACC AGATCCATCA AAACCAAGCA AGAGGTCATT
PRIMER 1F     ---------- ---------- ---------- ---------- ----------
PRIMER 1R     ---------- ---------- ---------- ---------- ----------
PRIMER 2F     ---------- ---------- ---------- ---------- ----------
PRIMER 2R     ---------- ---------- ---------- ---------- ----------
PRIMER 3F     ---------- ---------- ---------- ---------- ----------
PRIMER 3R     ---------- ---------- ---------- ---------- ----------
PRIMER 4F     ---------- ---------- ---------- ---------- ----------
PRIMER 4R     ---------- ---------- ---------- ---------- ----------
PRIMER 5F     ---------- ---------- ---------- ---------- ----------
PRIMER 5R     ---------- ---------- ---------- ---------- ----------
PRIMER 6F     ---------- ---------- ---------- ---------- ----------
PRIMER 6R     ---------- ---------- ---------- ---------- ----------
PRIMER 7R     ---------- ---------- ---------- ---------- ----------
PRIMER 8F     ---------- ---------- ---------- ---------- ----------

                    2460       2470       2480       2490       2500
              ....|....| ....|....| ....|....| ....|....| ....|....|
Wuhan-Hu-1    TATTGAAGAT CTACTTTTCA ACAAAGTGAC ACTTGCAGAT GCTGGCTTCA
B.1.1.7       TATTGAAGAT CTACTTTTCA ACAAAGTGAC ACTTGCAGAT GCTGGCTTCA
B.1.351       TATTGAAGAT CTACTTTTCA ACAAAGTGAC ACTTGCAGAT GCTGGCTTCA
P.1           TATTGAAGAT CTACTTTTCA ACAAAGTGAC ACTTGCAGAT GCTGGCTTCA
B.1.617       TATTGAAGAT CTACTTTTCA ACAAAGTGAC ACTTGCAGAT GCTGGCTTCA
PRIMER 1F     ---------- ---------- ---------- ---------- ----------
PRIMER 1R     ---------- ---------- ---------- ---------- ----------
PRIMER 2F     ---------- ---------- ---------- ---------- ----------
PRIMER 2R     ---------- ---------- ---------- ---------- ----------
PRIMER 3F     ---------- ---------- ---------- ---------- ----------
PRIMER 3R     ---------- ---------- ---------- ---------- ----------
PRIMER 4F     ---------- ---------- ---------- ---------- ----------
PRIMER 4R     ---------- ---------- ---------- ---------- ----------
PRIMER 5F     ---------- ---------- ---------- ---------- ----------
PRIMER 5R     ---------- ---------- ---------- ---------- ----------
PRIMER 6F     ---------- ---------- ---------- ---------- ----------
PRIMER 6R     ---------- ---------- ---------- ---------- ----------
PRIMER 7R     ---------- ---------- ---------- ---------- ----------
PRIMER 8F     ---------- ---------- ---------- ---------- ----------

                    2510       2520       2530       2540       2550
              ....|....| ....|....| ....|....| ....|....| ....|....|
Wuhan-Hu-1    TCAAACAATA TGGTGATTGC CTTGGTGATA TTGCTGCTAG AGACCTCATT
B.1.1.7       TCAAACAATA TGGTGATTGC CTTGGTGATA TTGCTGCTAG AGACCTCATT
B.1.351       TCAAACAATA TGGTGATTGC CTTGGTGATA TTGCTGCTAG AGACCTCATT
P.1           TCAAACAATA TGGTGATTGC CTTGGTGATA TTGCTGCTAG AGACCTCATT
B.1.617       TCAAACAATA TGGTGATTGC CTTGGTGATA TTGCTGCTAG AGACCTCATT
PRIMER 1F     ---------- ---------- ---------- ---------- ----------
PRIMER 1R     ---------- ---------- ---------- ---------- ----------
PRIMER 2F     ---------- ---------- ---------- ---------- ----------
PRIMER 2R     ---------- ---------- ---------- ---------- ----------
```

```
PRIMER 3F     ---------- ---------- ---------- ---------- ----------
PRIMER 3R     ---------- ---------- ---------- ---------- ----------
PRIMER 4F     ---------- ---------- ---------- ---------- ----------
PRIMER 4R     ---------- ---------- ---------- ---------- ----------
PRIMER 5F     ---------- ---------- ---------- ---------- ----------
PRIMER 5R     ---------- ---------- ---------- ---------- ----------
PRIMER 6F     ---------- ---------- ---------- ---------- ----------
PRIMER 6R     ---------- ---------- ---------- ---------- ----------
PRIMER 7R     ---------- ---------- ---------- ---------- ----------
PRIMER 8F     ---------- ---------- ---------- ---------- ----------

                        2560       2570       2580       2590       2600
              ....|....| ....|....| ....|....| ....|....| ....|....|
Wuhan-Hu-1    TGTGCACAAA AGTTTAACGG CCTTACTGTT TTGCCACCTT TGCTCACAGA
B.1.1.7       TGTGCACAAA AGTTTAACGG CCTTACTGTT TTGCCACCTT TGCTCACAGA
B.1.351       TGTGCACAAA AGTTTAACGG CCTTACTGTT TTGCCACCTT TGCTCACAGA
P.1           TGTGCACAAA AGTTTAACGG CCTTACTGTT TTGCCACCTT TGCTCACAGA
B.1.617       TGTGCACAAA AGTTTAACGG CCTTACTGTT TTGCCACCTT TGCTCACAGA
PRIMER 1F     ---------- ---------- ---------- ---------- ----------
PRIMER 1R     ---------- ---------- ---------- ---------- ----------
PRIMER 2F     ---------- ---------- ---------- ---------- ----------
PRIMER 2R     ---------- ---------- ---------- ---------- ----------
PRIMER 3F     ---------- ---------- ---------- ---------- ----------
PRIMER 3R     ---------- ---------- ---------- ---------- ----------
PRIMER 4F     ---------- ---------- ---------- ---------- ----------
PRIMER 4R     ---------- ---------- ---------- ---------- ----------
PRIMER 5F     ---------- ---------- ---------- ---------- ----------
PRIMER 5R     ---------- ---------- ---------- ---------- ----------
PRIMER 6F     ---------- ---------- ---------- ---------- ----------
PRIMER 6R     ---------- ---------- ---------- ---------- ----------
PRIMER 7R     ---------- ---------- ---------- ---------- ----------
PRIMER 8F     ---------- ---------- ---------- ---------- ----------

                        2610       2620       2630       2640       2650
              ....|....| ....|....| ....|....| ....|....| ....|....|
Wuhan-Hu-1    TGAAATGATT GCTCAATACA CTTCTGCACT GTTAGCGGGT ACAATCACTT
B.1.1.7       TGAAATGATT GCTCAATACA CTTCTGCACT GTTAGCGGGT ACAATCACTT
B.1.351       TGAAATGATT GCTCAATACA CTTCTGCACT GTTAGCGGGT ACAATCACTT
P.1           TGAAATGATT GCTCAATACA CTTCTGCACT GTTAGCGGGT ACAATCACTT
B.1.617       TGAAATGATT GCTCAATACA CTTCTGCACT GTTAGCGGGT ACAATCACTT
PRIMER 1F     ---------- ---------- ---------- ---------- ----------
PRIMER 1R     ---------- ---------- ---------- ---------- ----------
PRIMER 2F     ---------- ---------- ---------- ---------- ----------
PRIMER 2R     ---------- ---------- ---------- ---------- ----------
PRIMER 3F     ---------- ---------- ---------- ---------- ----------
PRIMER 3R     ---------- ---------- ---------- ---------- ----------
PRIMER 4F     ---------- ---------- ---------- ---------- ----------
PRIMER 4R     ---------- ---------- ---------- ---------- ----------
PRIMER 5F     ---------- ---------- ---------- ---------- ----------
PRIMER 5R     ---------- ---------- ---------- ---------- ----------
PRIMER 6F     ---------- ---------- ---------- ---------- ----------
PRIMER 6R     ---------- ---------- ---------- ---------- ----------
PRIMER 7R     ---------- ---------- ---------- ---------- ----------
PRIMER 8F     ---------- ---------- ---------- ---------- ----------

                        2660       2670       2680       2690       2700
              ....|....| ....|....| ....|....| ....|....| ....|....|
Wuhan-Hu-1    CTGGTTGGAC CTTTGGTGCA GGTGCTGCAT TACAAATACC ATTTGCTATG
B.1.1.7       CTGGTTGGAC CTTTGGTGCA GGTGCTGCAT TACAAATACC ATTTGCTATG
B.1.351       CTGGTTGGAC CTTTGGTGCA GGTGCTGCAT TACAAATACC ATTTGCTATG
P.1           CTGGTTGGAC CTTTGGTGCA GGTGCTGCAT TACAAATACC ATTTGCTATG
B.1.617       CTGGTTGGAC CTTTGGTGCA GGTGCTGCAT TACAAATACC ATTTGCTATG
```

```
PRIMER 1F    ---------- ---------- ---------- ---------- ----------
PRIMER 1R    ---------- ---------- ---------- ---------- ----------
PRIMER 2F    ---------- ---------- ---------- ---------- ----------
PRIMER 2R    ---------- ---------- ---------- ---------- ----------
PRIMER 3F    ---------- ---------- ---------- ---------- ----------
PRIMER 3R    ---------- ---------- ---------- ---------- ----------
PRIMER 4F    ---------- ---------- ---------- ---------- ----------
PRIMER 4R    ---------- ---------- ---------- ---------- ----------
PRIMER 5F    ---------- ---------- ---------- ---------- ----------
PRIMER 5R    ---------- ---------- ---------- ---------- ----------
PRIMER 6F    ---------- ---------- ---------- ---------- ----------
PRIMER 6R    ---------- ---------- ---------- ---------- ----------
PRIMER 7R    ---------- ---------- ---------- ---------- ----------
PRIMER 8F    ---------- ---------- ---------- ---------- ----------

                     2710       2720       2730       2740       2750
             ....|....| ....|....| ....|....| ....|....| ....|....|
Wuhan-Hu-1   CAAATGGCTT ATAGGTTTAA TGGTATTGGA GTTACACAGA ATGTTCTCTA
B.1.1.7      CAAATGGCTT ATAGGTTTAA TGGTATTGGA GTTACACAGA ATGTTCTCTA
B.1.351      CAAATGGCTT ATAGGTTTAA TGGTATTGGA GTTACACAGA ATGTTCTCTA
P.1          CAAATGGCTT ATAGGTTTAA TGGTATTGGA GTTACACAGA ATGTTCTCTA
B.1.617      CAAATGGCTT ATAGGTTTAA TGGTATTGGA GTTACACAGA ATGTTCTCTA
PRIMER 1F    ---------- ---------- ---------- ---------- ----------
PRIMER 1R    ---------- ---------- ---------- ---------- ----------
PRIMER 2F    ---------- ---------- ---------- ---------- ----------
PRIMER 2R    ---------- ---------- ---------- ---------- ----------
PRIMER 3F    ---------- ---------- ---------- ---------- ----------
PRIMER 3R    ---------- ---------- ---------- ---------- ----------
PRIMER 4F    ---------- ---------- ---------- ---------- ----------
PRIMER 4R    ---------- ---------- ---------- ---------- ----------
PRIMER 5F    ---------- ---------- ---------- ---------- ----------
PRIMER 5R    ---------- ---------- ---------- ---------- ----------
PRIMER 6F    ---------- ---------- ---------- ---------- ----------
PRIMER 6R    ---------- ---------- ---------- ---------- ----------
PRIMER 7R    ---------- ---------- ---------- ---------- ----------
PRIMER 8F    ---------- ---------- ---------- ---------- ----------

                     2760       2770       2780       2790       2800
             ....|....| ....|....| ....|....| ....|....| ....|....|
Wuhan-Hu-1   TGAGAACCAA AAATTGATTG CCAACCAATT TAATAGTGCT ATTGGCAAAA
B.1.1.7      TGAGAACCAA AAATTGATTG CCAACCAATT TAATAGTGCT ATTGGCAAAA
B.1.351      TGAGAACCAA AAATTGATTG CCAACCAATT TAATAGTGCT ATTGGCAAAA
P.1          TGAGAACCAA AAATTGATTG CCAACCAATT TAATAGTGCT ATTGGCAAAA
B.1.617      TGAGAACCAA AAATTGATTG CCAACCAATT TAATAGTGCT ATTGGCAAAA
PRIMER 1F    ---------- ---------- ---------- ---------- ----------
PRIMER 1R    ---------- ---------- ---------- ---------- ----------
PRIMER 2F    ---------- ---------- ---------- ---------- ----------
PRIMER 2R    ---------- ---------- ---------- ---------- ----------
PRIMER 3F    ---------- ---------- ---------- ---------- ----------
PRIMER 3R    ---------- ---------- ---------- ---------- ----------
PRIMER 4F    ---------- ---------- ---------- ---------- ----------
PRIMER 4R    ---------- ---------- ---------- ---------- ----------
PRIMER 5F    ---------- ---------- ---------- ---------- ----------
PRIMER 5R    ---------- ---------- ---------- ---------- ----------
PRIMER 6F    ---------- ---------- ---------- ---------- ----------
PRIMER 6R    ---------- ---------- ---------- ---------- ----------
PRIMER 7R    ---------- ---------- ---------- ---------- ----------
PRIMER 8F    ---------- ---------- ---------- ---------- ----------

                     2810       2820       2830       2840       2850
             ....|....| ....|....| ....|....| ....|....| ....|....|
Wuhan-Hu-1   TTCAAGACTC ACTTTCTTCC ACAGCAAGTG CACTTGGAAA ACTTCAAGAT
```

```
B.1.1.7     TTCAAGACTC ACTTTCTTCC ACAGCAAGTG CACTTGGAAA ACTTCAAGAT
B.1.351     TTCAAGACTC ACTTTCTTCC ACAGCAAGTG CACTTGGAAA ACTTCAAGAT
P.1         TTCAAGACTC ACTTTCTTCC ACAGCAAGTG CACTTGGAAA ACTTCAAGAT
B.1.617     TTCAAGACTC ACTTTCTTCC ACAGCAAGTG CACTTGGAAA ACTTCAAAAT
PRIMER 1F   ---------- ---------- ---------- ---------- ----------
PRIMER 1R   ---------- ---------- ---------- ---------- ----------
PRIMER 2F   ---------- ---------- ---------- ---------- ----------
PRIMER 2R   ---------- ---------- ---------- ---------- ----------
PRIMER 3F   ---------- ---------- ---------- ---------- ----------
PRIMER 3R   ---------- ---------- ---------- ---------- ----------
PRIMER 4F   ---------- ---------- ---------- ---------- ----------
PRIMER 4R   ---------- ---------- ---------- ---------- ----------
PRIMER 5F   ----AGACTC ACTTTCTTCC ACAGCA---- ---------- ----------
PRIMER 5R   ---------- ---------- ---------- ---------- ----------
PRIMER 6F   ---------- ---------- ---------- ---------- ----------
PRIMER 6R   ---------- ---------- ---------- ---------- ----------
PRIMER 7R   ---------- ---------- ---------- ---------- ----------
PRIMER 8F   ---------- ---------- ---------- ---------- ----------

                    2860       2870       2880       2890       2900
            ....|....| ....|....| ....|....| ....|....| ....|....|
Wuhan-Hu-1  GTGGTCAACC AAAATGCACA AGCTTTAAAC ACGCTTGTTA AACAACTTAG
B.1.1.7     GTGGTCAACC AAAATGCACA AGCTTTAAAC ACGCTTGTTA AACAACTTAG
B.1.351     GTGGTCAACC AAAATGCACA AGCTTTAAAC ACGCTTGTTA AACAACTTAG
P.1         GTGGTCAACC AAAATGCACA AGCTTTAAAC ACGCTTGTTA AACAACTTAG
B.1.617     GTGGTCAACC AAAATGCACA AGCTTTAAAC ACGCTTGTTA AACAACTTAG
PRIMER 1F   ---------- ---------- ---------- ---------- ----------
PRIMER 1R   ---------- ---------- ---------- ---------- ----------
PRIMER 2F   ---------- ---------- ---------- ---------- ----------
PRIMER 2R   ---------- ---------- ---------- ---------- ----------
PRIMER 3F   ---------- ---------- ---------- ---------- ----------
PRIMER 3R   ---------- ---------- ---------- ---------- ----------
PRIMER 4F   ---------- ---------- ---------- ---------- ----------
PRIMER 4R   --GGTCAACC AAAATGCACA AGC------- ---------- ----------
PRIMER 5F   ---------- ---------- ---------- ---------- ----------
PRIMER 5R   ---------- ---------- ---------- ---------- ----------
PRIMER 6F   ---------- ---------- ---------- ---------- ----------
PRIMER 6R   ---------- ---------- ---------- ---------- ----------
PRIMER 7R   ---------- ---------- ---------- ---------- ----------
PRIMER 8F   ---------- ---------- ---------- ---------- ----------

                    2910       2920       2930       2940       2950
            ....|....| ....|....| ....|....| ....|....| ....|....|
Wuhan-Hu-1  CTCCAATTTT GGTGCAATTT CAAGTGTTTT AAATGATATC CTTTCACGTC
B.1.1.7     CTCCAATTTT GGTGCAATTT CAAGTGTTTT AAATGATATC CTTGCACGTC
B.1.351     CTCCAATTTT GGTGCAATTT CAAGTGTTTT AAATGATATC CTTTCACGTC
P.1         CTCCAATTTT GGTGCAATTT CAAGTGTTTT AAATGATATC CTTTCACGTC
B.1.617     CTCCAATTTT GGTGCAATTT CAAGTGTTTT AAATGATATC CTTTCACGTC
PRIMER 1F   ---------- ---------- ---------- ---------- ----------
PRIMER 1R   ---------- ---------- ---------- ---------- ----------
PRIMER 2F   ---------- ---------- ---------- ---------- ----------
PRIMER 2R   ---------- ---------- ---------- ---------- ----------
PRIMER 3F   ---------- ---------- ---------- ---------- ----------
PRIMER 3R   ---------- ---------- ---------- ---------- ----------
PRIMER 4F   ---------- ---------- ---------- ---------- ----------
PRIMER 4R   ---------- ---------- ---------- ---------- ----------
PRIMER 5F   ---------- ---------- ---------- ---------- ----------
PRIMER 5R   ---------- ---------- ---------- ---------- ----------
PRIMER 6F   ---------- ---------- ---------- ---------- ----------
PRIMER 6R   ---------- ---------- ---------- ---------- ----------
PRIMER 7R   ---------- ---------- ---------- ---------- ----------
PRIMER 8F   ---------- ---------- ---------- ---------- ----------
```

```
                    2960       2970       2980       2990       3000
            ....|....| ....|....| ....|....| ....|....| ....|....|
Wuhan-Hu-1  TTGACAAAGT TGAGGCTGAA GTGCAAATTG ATAGGTTGAT CACAGGCAGA
B.1.1.7     TTGACAAAGT TGAGGCTGAA GTGCAAATTG ATAGGTTGAT CACAGGCAGA
B.1.351     TTGACAAAGT TGAGGCTGAA GTGCAAATTG ATAGGTTGAT CACAGGCAGA
P.1         TTGACAAAGT TGAGGCTGAA GTGCAAATTG ATAGGTTGAT CACAGGCAGA
B.1.617     TTGACAAAGT TGAGGCTGAA GTGCAAATTG ATAGGTTGAT CACAGGCAGA
PRIMER 1F   ---------- ---------- ---------- ---------- ----------
PRIMER 1R   ---------- ---------- ---------- ---------- ----------
PRIMER 2F   ---------- ---------- ---------- ---------- ----------
PRIMER 2R   ---------- ---------- ---------- ---------- ----------
PRIMER 3F   ---------- ---------- ---------- ---------- ----------
PRIMER 3R   ---------- ---------- ---------- ---------- ----------
PRIMER 4F   ---------- ---------- ---------- ---------- ----------
PRIMER 4R   ---------- ---------- ---------- ---------- ----------
PRIMER 5F   ---------- ---------- ---------- ---------- ----------
PRIMER 5R   ---------- ---------- ---------- ---------- ----------
PRIMER 6F   ---------- ---------- ---------- ---------- ----------
PRIMER 6R   ---------- ---------- ---------- ---------- ----------
PRIMER 7R   ---------- ---------- ---------- ---------- ----------
PRIMER 8F   ---------- ---------- ---------- ---------- ----------

                    3010       3020       3030       3040       3050
            ....|....| ....|....| ....|....| ....|....| ....|....|
Wuhan-Hu-1  CTTCAAAGTT TGCAGACATA TGTGACTCAA CAATTAATTA GAGCTGCAGA
B.1.1.7     CTTCAAAGTT TGCAGACATA TGTGACTCAA CAATTAATTA GAGCTGCAGA
B.1.351     CTTCAAAGTT TGCAGACATA TGTGACTCAA CAATTAATTA GAGCTGCAGA
P.1         CTTCAAAGTT TGCAGACATA TGTGACTCAA CAATTAATTA GAGCTGCAGA
B.1.617     CTTCAAAGTT TGCAGACATA TGTGACTCAA CAATTAATTA GAGCTGCAGA
PRIMER 1F   ---------- ---------- ---------- ---------- ----------
PRIMER 1R   ---------- ---------- ---------- ---------- ----------
PRIMER 2F   ---------- ---------- ---------- ---------- ----------
PRIMER 2R   ---------- ---------- ---------- ---------- ----------
PRIMER 3F   ---------- ---------- ---------- ---------- ----------
PRIMER 3R   ---------- ---------- ---------- ---------- ----------
PRIMER 4F   ---------- ---------- ---------- ---------- ----------
PRIMER 4R   ---------- ---------- ---------- ---------- ----------
PRIMER 5F   ---------- ---------- ---------- ---------- ----------
PRIMER 5R   ---------- ---------- ---------- ---------- ----------
PRIMER 6F   ---------- ---------- ---------- ---------- ----------
PRIMER 6R   ---------- ---------- ---------- ---------- ----------
PRIMER 7R   ---------- ---------- ---------- ---------- ----------
PRIMER 8F   ---------- ---------- ---------- ---------- ----------

                    3060       3070       3080       3090       3100
            ....|....| ....|....| ....|....| ....|....| ....|....|
Wuhan-Hu-1  AATCAGAGCT TCTGCTAATC TTGCTGCTAC TAAAATGTCA GAGTGTGTAC
B.1.1.7     AATCAGAGCT TCTGCTAATC TTGCTGCTAC TAAAATGTCA GAGTGTGTAC
B.1.351     AATCAGAGCT TCTGCTAATC TTGCTGCTAC TAAAATGTCA GAGTGTGTAC
P.1         AATCAGAGCT TCTGCTAATC TTGCTGCTAT TAAAATGTCA GAGTGTGTAC
B.1.617     AATCAGAGCT TCTGCTAATC TTGCTGCTAC TAAAATGTCA GAGTGTGTAC
PRIMER 1F   ---------- ---------- ---------- ---------- ----------
PRIMER 1R   ---------- ---------- ---------- ---------- ----------
PRIMER 2F   ---------- ---------- ---------- ---------- ----------
PRIMER 2R   ---------- ---------- ---------- ---------- ----------
PRIMER 3F   ---------- ---------- ---------- ---------- ----------
PRIMER 3R   ---------- ---------- ---------- ---------- ----------
PRIMER 4F   ---------- ---------- ---------- ---------- ----------
PRIMER 4R   ---------- ---------- ---------- ---------- ----------
PRIMER 5F   ---------- ---------- ---------- ---------- ----------
PRIMER 5R   ---------- ---------- ---------- ---------- ----------
```

```
PRIMER 6F    ---------T TCTGCTAATC TTGCTGCTAC T--------- ----------
PRIMER 6R    ---------- ---------- ---------- ---------- ----------
PRIMER 7R    ---------- ---------- ---------- ---------- ----------
PRIMER 8F    ---------T TCTGCTAATC TTGCTGCTAC T--------- ----------

                      3110       3120       3130       3140       3150
             ....|....| ....|....| ....|....| ....|....| ....|....|
Wuhan-Hu-1   TTGGACAATC AAAAAGAGTT GATTTTTGTG GAAAGGGCTA TCATCTTATG
B.1.1.7      TTGGACAATC AAAAAGAGTT GATTTTTGTG GAAAGGGCTA TCATCTTATG
B.1.351      TTGGACAATC AAAAAGAGTT GATTTTTGTG GAAAGGGCTA TCATCTTATG
P.1          TTGGACAATC AAAAAGAGTT GATTTTTGTG GAAAGGGCTA TCATCTTATG
B.1.617      TTGGACAATC AAAAAGAGTT GATTTTTGTG GAAAGGGCTA TCATCTTATG
PRIMER 1F    ---------- ---------- ---------- ---------- ----------
PRIMER 1R    ---------- ---------- ---------- ---------- ----------
PRIMER 2F    ---------- ---------- ---------- ---------- ----------
PRIMER 2R    ---------- ---------- ---------- ---------- ----------
PRIMER 3F    ---------- ---------- ---------- ---------- ----------
PRIMER 3R    ---------- ---------- ---------- ---------- ----------
PRIMER 4F    ---------- ---------- ---------- ---------- ----------
PRIMER 4R    ---------- ---------- ---------- ---------- ----------
PRIMER 5F    ---------- ---------- ---------- ---------- ----------
PRIMER 5R    ---------- ---------- ------TGTG GAAAGGGCTA TCATCT----
PRIMER 6F    ---------- ---------- ---------- ---------- ----------
PRIMER 6R    ---------- ---------- ---------- ---------- ----------
PRIMER 7R    ---------- ---------- ---------- ---------- ----------
PRIMER 8F    ---------- ---------- ---------- ---------- ----------

                      3160       3170       3180       3190       3200
             ....|....| ....|....| ....|....| ....|....| ....|....|
Wuhan-Hu-1   TCCTTCCCTC AGTCAGCACC TCATGGTGTA GTCTTCTTGC ATGTGACTTA
B.1.1.7      TCCTTCCCTC AGTCAGCACC TCATGGTGTA GTCTTCTTGC ATGTGACTTA
B.1.351      TCCTTCCCTC AGTCAGCACC TCATGGTGTA GTCTTCTTGC ATGTGACTTA
P.1          TCCTTCCCTC AGTCAGCACC TCATGGTGTA GTCTTCTTGC ATGTGACTTA
B.1.617      TCCTTCCCTC AGTCAGCACC TCATGGTGTA GTCTTCTTGC ATGTGACTTA
PRIMER 1F    ---------- ---------- ---------- ---------- ----------
PRIMER 1R    ---------- ---------- ---------- ---------- ----------
PRIMER 2F    ---------- ---------- ---------- ---------- ----------
PRIMER 2R    ---------- ---------- ---------- ---------- ----------
PRIMER 3F    ---------- ---------- ---------- ---------- ----------
PRIMER 3R    ---------- ---------- ---------- ---------- ----------
PRIMER 4F    ---------- ---------- ---------- ---------- ----------
PRIMER 4R    ---------- ---------- ---------- ---------- ----------
PRIMER 5F    ---------- ---------- ---------- ---------- ----------
PRIMER 5R    ---------- ---------- ---------- ---------- ----------
PRIMER 6F    ---------- ---------- ---------- ---------- ----------
PRIMER 6R    ---------- ---------- ---------- ---------- ----------
PRIMER 7R    ---------- ---------- ---------- ---------- ----------
PRIMER 8F    ---------- ---------- ---------- ---------- ----------

                      3210       3220       3230       3240       3250
             ....|....| ....|....| ....|....| ....|....| ....|....|
Wuhan-Hu-1   TGTCCCTGCA CAAGAAAAGA ACTTCACAAC TGCTCCTGCC ATTTGTCATG
B.1.1.7      TGTCCCTGCA CAAGAAAAGA ACTTCACAAC TGCTCCTGCC ATTTGTCATG
B.1.351      TGTCCCTGCA CAAGAAAAGA ACTTCACAAC TGCTCCTGCC ATTTGTCATG
P.1          TGTCCCTGCA CAAGAAAAGA ACTTCACAAC TGCTCCTGCC ATTTGTCATG
B.1.617      TGTCCCTGCA CAAGAAAAGA ACTTCACAAC TGCTCCTGCC ATTTGTCATG
PRIMER 1F    ---------- ---------- ---------- ---------- ----------
PRIMER 1R    ---------- ---------- ---------- ---------- ----------
PRIMER 2F    ---------- ---------- ---------- ---------- ----------
PRIMER 2R    ---------- ---------- ---------- ---------- ----------
PRIMER 3F    ---------- ---------- ---------- ---------- ----------
PRIMER 3R    ---------- ---------- ---------- ---------- ----------
```

```
PRIMER 4F    ---------- ---------- ---------- ---------- ----------
PRIMER 4R    ---------- ---------- ---------- ---------- ----------
PRIMER 5F    ---------- ---------- ---------- ---------- ----------
PRIMER 5R    ---------- ---------- ---------- ---------- ----------
PRIMER 6F    ---------- ---------- ---------- ---------- ----------
PRIMER 6R    ---------- ---------- ---------- ---------- ----------
PRIMER 7R    ---------- ---------- ---------- ---------- ----------
PRIMER 8F    ---------- ---------- ---------- ---------- ----------

                   3260       3270       3280       3290       3300
             ....|....| ....|....| ....|....| ....|....| ....|....|
Wuhan-Hu-1   ATGGAAAAGC ACACTTTCCT CGTGAAGGTG TCTTTGTTTC AAATGGCACA
B.1.1.7      ATGGAAAAGC ACACTTTCCT CGTGAAGGTG TCTTTGTTTC AAATGGCACA
B.1.351      ATGGAAAAGC ACACTTTCCT CGTGAAGGTG TCTTTGTTTC AAATGGCACA
P.1          ATGGAAAAGC ACACTTTCCT CGTGAAGGTG TCTTTGTTTC AAATGGCACA
B.1.617      ATGGAAAAGC ACACTTTCCT CGTGAAGGTG TCTTTGTTTC AAATGGCACA
PRIMER 1F    ---------- ---------- ---------- ---------- ----------
PRIMER 1R    ---------- ---------- ---------- ---------- ----------
PRIMER 2F    ---------- ---------- ---------- ---------- ----------
PRIMER 2R    ---------- ---------- ---------- ---------- ----------
PRIMER 3F    ---------- ---------- ---------- ---------- ----------
PRIMER 3R    ---------- ---------- ---------- ---------- ----------
PRIMER 4F    ---------- ---------- ---------- ---------- ----------
PRIMER 4R    ---------- ---------- ---------- ---------- ----------
PRIMER 5F    ---------- ---------- ---------- ---------- ----------
PRIMER 5R    ---------- ---------- ---------- ---------- ----------
PRIMER 6F    ---------- ---------- ---------- ---------- ----------
PRIMER 6R    ---------- ---------- ---------- ---------- ----------
PRIMER 7R    ---------- ---------- ---------- ---------- ----------
PRIMER 8F    ---------- ---------- ---------- ---------- ----------

                   3310       3320       3330       3340       3350
             ....|....| ....|....| ....|....| ....|....| ....|....|
Wuhan-Hu-1   CACTGGTTTG TAACACAAAG GAATTTTTAT GAACCACAAA TCATTACTAC
B.1.1.7      CACTGGTTTG TAACACAAAG GAATTTTTAT GAACCACAAA TCATTACTAC
B.1.351      CACTGGTTTG TAACACAAAG GAATTTTTAT GAACCACAAA TCATTACTAC
P.1          CACTGGTTTG TAACACAAAG GAATTTTTAT GAACCACAAA TCATTACTAC
B.1.617      CACTGGTTTG TAACACAAAG GAATTTTTAT GAACCACAAA TCATTACTAC
PRIMER 1F    ---------- ---------- ---------- ---------- ----------
PRIMER 1R    ---------- ---------- ---------- ---------- ----------
PRIMER 2F    ---------- ---------- ---------- ---------- ----------
PRIMER 2R    ---------- ---------- ---------- ---------- ----------
PRIMER 3F    ---------- ---------- ---------- ---------- ----------
PRIMER 3R    ---------- ---------- ---------- ---------- ----------
PRIMER 4F    ---------- ---------- ---------- ---------- ----------
PRIMER 4R    ---------- ---------- ---------- ---------- ----------
PRIMER 5F    ---------- ---------- ---------- ---------- ----------
PRIMER 5R    ---------- ---------- ---------- ---------- ----------
PRIMER 6F    ---------- ---------- ---------- ---------- ----------
PRIMER 6R    ---------- ---------- ---------- ---------- ----------
PRIMER 7R    ---------- ---------- ---------- ---------- ----------
PRIMER 8F    ---------- ---------- ---------- ---------- ----------

                   3360       3370       3380       3390       3400
             ....|....| ....|....| ....|....| ....|....| ....|....|
Wuhan-Hu-1   AGACAACACA TTTGTGTCTG GTAACTGTGA TGTTGTAATA GGAATTGTCA
B.1.1.7      ACACAACACA TTTGTGTCTG GTAACTGTGA TGTTGTAATA GGAATTGTCA
B.1.351      AGACAACACA TTTGTGTCTG GTAACTGTGA TGTTGTAATA GGAATTGTCA
P.1          AGACAACACA TTTGTGTCTG GTAACTGTGA TGTTGTAATA GGAATTGTCA
B.1.617      AGACAACACA TTTGTGTCTG GTAACTGTGA TGTTGTAATA GGAATTGTCA
PRIMER 1F    ---------- ---------- ---------- ---------- ----------
PRIMER 1R    ---------- ---------- ---------- ---------- ----------
```

```
PRIMER 2F     ---------- ---------- ---------- ---------- ----------
PRIMER 2R     ---------- ---------- ---------- ---------- ----------
PRIMER 3F     ---------- ---------- ---------- ---------- ----------
PRIMER 3R     ---------- ---------- ---------- ---------- ----------
PRIMER 4F     ---------- ---------- ---------- ---------- ----------
PRIMER 4R     ---------- ---------- ---------- ---------- ----------
PRIMER 5F     ---------- ---------- ---------- ---------- ----------
PRIMER 5R     ---------- ---------- ---------- ---------- ----------
PRIMER 6F     ---------- ---------- ---------- ---------- ----------
PRIMER 6R     ---------- ---------- ---------- ---------- ----------
PRIMER 7R     ---------- ---------- ---------- ---------- ----------
PRIMER 8F     ---------- ---------- ---------- ---------- ----------

                       3410       3420       3430       3440       3450
              ....|....| ....|....| ....|....| ....|....| ....|....|
Wuhan-Hu-1    ACAACACAGT TTATGATCCT TTGCAACCTG AATTAGACTC ATTCAAGGAG
B.1.1.7       ACAACACAGT TTATGATCCT TTGCAACCTG AATTAGACTC ATTCAAGGAG
B.1.351       ACAACACAGT TTATGATCCT TTGCAACCTG AATTAGACTC ATTCAAGGAG
P.1           ACAACACAGT TTATGATCCT TTGCAACCTG AATTAGACTC ATTCAAGGAG
B.1.617       ACAACACAGT TTATGATCCT TTGCAACCTG AATTAGACTC ATTCAAGGAG
PRIMER 1F     ---------- ---------- ---------- ---------- ----------
PRIMER 1R     ---------- ---------- ---------- ---------- ----------
PRIMER 2F     ---------- ---------- ---------- ---------- ----------
PRIMER 2R     ---------- ---------- ---------- ---------- ----------
PRIMER 3F     ---------- ---------- ---------- ---------- ----------
PRIMER 3R     ---------- ---------- ---------- ---------- ----------
PRIMER 4F     ---------- ---------- ---------- ---------- ----------
PRIMER 4R     ---------- ---------- ---------- ---------- ----------
PRIMER 5F     ---------- ---------- ---------- ---------- ----------
PRIMER 5R     ---------- ---------- ---------- ---------- ----------
PRIMER 6F     ---------- ---------- ---------- ---------- ----------
PRIMER 6R     ---------- ---------- ---------- ---------- ----------
PRIMER 7R     ---------- ---------- ---------- ---------- ----------
PRIMER 8F     ---------- ---------- ---------- ---------- ----------

                       3460       3470       3480       3490       3500
              ....|....| ....|....| ....|....| ....|....| ....|....|
Wuhan-Hu-1    GAGTTAGATA AATATTTTAA GAATCATACA TCACCAGATG TTGATTTAGG
B.1.1.7       GAGTTAGATA AATATTTTAA GAATCATACA TCACCAGATG TTGATTTAGG
B.1.351       GAGTTAGATA AATATTTTAA GAATCATACA TCACCAGATG TTGATTTAGG
P.1           GAGTTAGATA AATATTTTAA GAATCATACA TCACCAGATG TTGATTTAGG
B.1.617       GAGTTAGATA AATATTTTAA GAATCATACA TCACCAGATG TTGATTTAGG
PRIMER 1F     ---------- ---------- ---------- ---------- ----------
PRIMER 1R     ---------- ---------- ---------- ---------- ----------
PRIMER 2F     ---------- ---------- ---------- ---------- ----------
PRIMER 2R     ---------- ---------- ---------- ---------- ----------
PRIMER 3F     ---------- ---------- ---------- ---------- ----------
PRIMER 3R     ---------- ---------- ---------- ---------- ----------
PRIMER 4F     ---------- ---------- ---------- ---------- ----------
PRIMER 4R     ---------- ---------- ---------- ---------- ----------
PRIMER 5F     ---------- ---------- ---------- ---------- ----------
PRIMER 5R     ---------- ---------- ---------- ---------- ----------
PRIMER 6F     ---------- ---------- ---------- ---------- ----------
PRIMER 6R     ---------- ---------- ---------- ---------- ----------
PRIMER 7R     ---------- ---------- ---------- ---------- ----------
PRIMER 8F     ---------- ---------- ---------- ---------- ----------

                       3510       3520       3530       3540       3550
              ....|....| ....|....| ....|....| ....|....| ....|....|
Wuhan-Hu-1    TGACATCTCT GGCATTAATG CTTCAGTTGT AAACATTCAA AAAGAAATTG
B.1.1.7       TGACATCTCT GGCATTAATG CTTCAGTTGT AAACATTCAA AAAGAAATTG
B.1.351       TGACATCTCT GGCATTAATG CTTCAGTTGT AAACATTCAA AAAGAAATTG
```

```
P.1          TGACATCTCT GGCATTAATG CTTCATTTGT AAACATTCAA AAAGAAATTG
B.1.617      TGACATCTCT GGCATTAATG CTTCAGTTGT AAACATTCAA AAAGAAATTG
PRIMER 1F    ---------- ---------- ---------- ---------- ----------
PRIMER 1R    ---------- ---------- ---------- ---------- ----------
PRIMER 2F    ---------- ---------- ---------- ---------- ----------
PRIMER 2R    ---------- ---------- ---------- ---------- ----------
PRIMER 3F    ---------- ---------- ---------- ---------- ----------
PRIMER 3R    ---------- ---------- ---------- ---------- ----------
PRIMER 4F    ---------- ---------- ---------- ---------- ----------
PRIMER 4R    ---------- ---------- ---------- ---------- ----------
PRIMER 5F    ---------- ---------- ---------- ---------- ----------
PRIMER 5R    ---------- ---------- ---------- ---------- ----------
PRIMER 6F    ---------- ---------- ---------- ---------- ----------
PRIMER 6R    ---------- ---------- ---------- ---------- ----------
PRIMER 7R    ---------- ---------- ---------- ---------- ----------
PRIMER 8F    ---------- ---------- ---------- ---------- ----------

                   3560       3570       3580       3590       3600
             ....|....| ....|....| ....|....| ....|....| ....|....|
Wuhan-Hu-1   ACCGCCTCAA TGAGGTTGCC AAGAATTTAA ATGAATCTCT CATCGATCTC
B.1.1.7      ACCGCCTCAA TGAGGTTGCC AAGAATTTAA ATGAATCTCT CATCGATCTC
B.1.351      ACCGCCTCAA TGAGGTTGCC AAGAATTTAA ATGAATCTCT CATCGATCTC
P.1          ACCGCCTCAA TGAGGTTGCC AAGAATTTAA ATGAATCTCT CATCGATCTC
B.1.617      ACCGCCTCAA TGAGGTTGCC AAGAATTTAA ATGAATCTCT CATCGATCTC
PRIMER 1F    ---------- ---------- ---------- ---------- ----------
PRIMER 1R    ---------- ---------- ---------- ---------- ----------
PRIMER 2F    ---------- ---------- ---------- ---------- ----------
PRIMER 2R    ---------- ---------- ---------- ---------- ----------
PRIMER 3F    ---------- ---------- ---------- ---------- ----------
PRIMER 3R    ---------- ---------- ---------- ---------- ----------
PRIMER 4F    ---------- ---------- ---------- ---------- ----------
PRIMER 4R    ---------- ---------- ---------- ---------- ----------
PRIMER 5F    ---------- ---------- ---------- ---------- ----------
PRIMER 5R    ---------- ---------- ---------- ---------- ----------
PRIMER 6F    ---------- ---------- ---------- ---------- ----------
PRIMER 6R    ---------- ---------- ---------- ---------- ----------
PRIMER 7R    ---------- ---------- ---------- ---------- ----------
PRIMER 8F    ---------- ---------- ---------- ---------- ----------

                   3610       3620       3630       3640       3650
             ....|....| ....|....| ....|....| ....|....| ....|....|
Wuhan-Hu-1   CAAGAACTTG GAAAGTATGA GCAGTATATA AAATGGCCAT GGTACATTTG
B.1.1.7      CAAGAACTTG GAAAGTATGA GCAGTATATA AAATGGCCAT GGTACATTTG
B.1.351      CAAGAACTTG GAAAGTATGA GCAGTATATA AAATGGCCAT GGTACATTTG
P.1          CAAGAACTTG GAAAGTATGA GCAGTATATA AAATGGCCAT GGTACATTTG
B.1.617      CAAGAACTTG GAAAGTATGA GCAGTATATA AAATGGCCAT GGTACATTTG
PRIMER 1F    ---------- ---------- ---------- ---------- ----------
PRIMER 1R    ---------- ---------- ---------- ---------- ----------
PRIMER 2F    ---------- ---------- ---------- ---------- ----------
PRIMER 2R    ---------- ---------- ---------- ---------- ----------
PRIMER 3F    ---------- ---------- ---------- ---------- ----------
PRIMER 3R    ---------- ---------- ---------- ---------- ----------
PRIMER 4F    ---------- ---------- ---------- ---------- ----------
PRIMER 4R    ---------- ---------- ---------- ---------- ----------
PRIMER 5F    ---------- ---------- ---------- ---------- ----------
PRIMER 5R    ---------- ---------- ---------- ---------- ----------
PRIMER 6F    ---------- ---------- ---------- ---------- ----------
PRIMER 6R    ---------- ---------- ---------- ---------- ----------
PRIMER 7R    ---------- ---------- ---------- ---------- ----------
PRIMER 8F    ---------- ---------- ---------- ---------- ----------

                   3660       3670       3680       3690       3700
```

```
              ....|....| ....|....| ....|....| ....|....| ....|....|
Wuhan-Hu-1    GCTAGGTTTT ATAGCTGGCT TGATTGCCAT AGTAATGGTG ACAATTATGC
B.1.1.7       GCTAGGTTTT ATAGCTGGCT TGATTGCCAT AGTAATGGTG ACAATTATGC
B.1.351       GCTAGGTTTT ATAGCTGGCT TGATTGCCAT AGTAATGGTG ACAATTATGC
P.1           GCTAGGTTTT ATAGCTGGCT TGATTGCCAT AGTAATGGTG ACAATTATGC
B.1.617       GCTAGGTTTT ATAGCTGGCT TGATTGCCAT AGTAATGGTG ACAATTATGC
PRIMER 1F     ---------- ---------- ---------- ---------- ----------
PRIMER 1R     ---------- ---------- ---------- ---------- ----------
PRIMER 2F     ---------- ---------- ---------- ---------- ----------
PRIMER 2R     ---------- ---------- ---------- ---------- ----------
PRIMER 3F     ---------- ---------- ---------- ---------- ----------
PRIMER 3R     ---------- ---------- ---------- ---------- ----------
PRIMER 4F     ---------- ---------- ---------- ---------- ----------
PRIMER 4R     ---------- ---------- ---------- ---------- ----------
PRIMER 5F     ---------- ---------- ---------- ---------- ----------
PRIMER 5R     ---------- ---------- ---------- ---------- ----------
PRIMER 6F     ---------- ---------- ---------- ---------- ----------
PRIMER 6R     ---------- ---------- ---------- ---------- ----------
PRIMER 7R     ---------- ---------- ---------- ---------- ----------
PRIMER 8F     ---------- ---------- ---------- ---------- ----------

                       3710       3720       3730       3740       3750
              ....|....| ....|....| ....|....| ....|....| ....|....|
Wuhan-Hu-1    TTTGCTGTAT GACCAGTTGC TGTAGTTGTC TCAAGGGCTG TTGTTCTTGT
B.1.1.7       TTTGCTGTAT GACCAGTTGC TGTAGTTGTC TCAAGGGCTG TTGTTCTTGT
B.1.351       TTTGCTGTAT GACCAGTTGC TGTAGTTGTC TCAAGGGCTG TTGTTCTTGT
P.1           TTTGCTGTAT GACCAGTTGC TGTAGTTGTC TCAAGGGCTG TTGTTCTTGT
B.1.617       TTTGCTGTAT GACCAGTTGC TGTAGTTGTC TCAAGGGCTG TTGTTCTTGT
PRIMER 1F     ---------- ---------- ---------- ---------- ----------
PRIMER 1R     ---------- ---------- ---------- ---------- ----------
PRIMER 2F     ---------- ---------- ---------- ---------- ----------
PRIMER 2R     ---------- ---------- ---------- ---------- ----------
PRIMER 3F     ---------- ---------- ---------- ---------- ----------
PRIMER 3R     ---------- ---------- ---------- ---------- ----------
PRIMER 4F     ---------- ---------- ---------- ---------- ----------
PRIMER 4R     ---------- ---------- ---------- ---------- ----------
PRIMER 5F     ---------- ---------- ---------- ---------- ----------
PRIMER 5R     ---------- ---------- ---------- ---------- ----------
PRIMER 6F     ---------- ---------- ---------- ---------- ----------
PRIMER 6R     ---------- ---------- ---------- ---------- ----------
PRIMER 7R     ---------- ---------- ---------- ---------- ----------
PRIMER 8F     ---------- ---------- ---------- ---------- ----------

                       3760       3770       3780       3790       3800
              ....|....| ....|....| ....|....| ....|....| ....|....|
Wuhan-Hu-1    GGATCCTGCT GCAAATTTGA TGAAGACGAC TCTGAGCCAG TGCTCAAAGG
B.1.1.7       GGATCCTGCT GCAAATTTGA TGAAGACGAC TCTGAGCCAG TGCTCAAAGG
B.1.351       GGATCCTGCT GCAAATTTGA TGAAGACGAC TCTGAGCCAG TGCTCAAAGG
P.1           GGATCCTGCT GCAAATTTGA TGAAGACGAC TCTGAGCCAG TGCTCAAAGG
B.1.617       GGATCCTGCT GCAAATTTGA TGAAGACGAC TCTGAGCCAG TGCTCAAAGG
PRIMER 1F     ---------- ---------- ---------- ---------- ----------
PRIMER 1R     ---------- ---------- ---------- ---------- ----------
PRIMER 2F     ---------- ---------- ---------- ---------- ----------
PRIMER 2R     ---------- ---------- ---------- ---------- ----------
PRIMER 3F     ---------- ---------- ---------- ---------- ----------
PRIMER 3R     ---------- ---------- ---------- ---------- ----------
PRIMER 4F     ---------- ---------- ---------- ---------- ----------
PRIMER 4R     ---------- ---------- ---------- ---------- ----------
PRIMER 5F     ---------- ---------- ---------- ---------- ----------
PRIMER 5R     ---------- ---------- ---------- ---------- ----------
PRIMER 6F     ---------- ---------- ---------- ---------- ----------
PRIMER 6R     ---------- ---------- ---------- ---------- --------GG
```

```
PRIMER 7R    ---------- ---------- ---------- ---------- ----------
PRIMER 8F    ---------- ---------- ---------- ---------- ----------


               3810       3820
             ....|....| ....|....| ....
Wuhan-Hu-1   AGTCAAATTA CATTACACAT AA
B.1.1.7      AGTCAAATTA CATTACACAT AA
B.1.351      AGTCAAATTA CATTACACAT AA
P.1          AGTCAAATTA CATTACACAT AA
B.1.617      AGTCAAATTA CATTACACAT AA
PRIMER 1F    ---------- ---------- --
PRIMER 1R    ---------- ---------- --
PRIMER 2F    ---------- ---------- --
PRIMER 2R    ---------- ---------- --
PRIMER 3F    ---------- ---------- --
PRIMER 3R    ---------- ---------- --
PRIMER 4F    ---------- ---------- --
PRIMER 4R    ---------- ---------- --
PRIMER 5F    ---------- ---------- --
PRIMER 5R    ---------- ---------- --
PRIMER 6F    ---------- ---------- --
PRIMER 6R    AGTCAAATTA CATTACACAT AAAC
PRIMER 7R    ---------- ---------- --
PRIMER 8F    ---------- ---------- --
```
