# Supplementary figures and images for: Genomic surveillance of SARS-CoV-2 Spike gene by sanger sequencing
Source: PLoS One. 2022 Jan 20;17(1):e0262170. doi: 10.1371/journal.pone.0262170 (PMC8775319; doi:10.1371/journal.pone.0262170)

S3 Fig: Flowchart describing the sequential steps of the protocol.

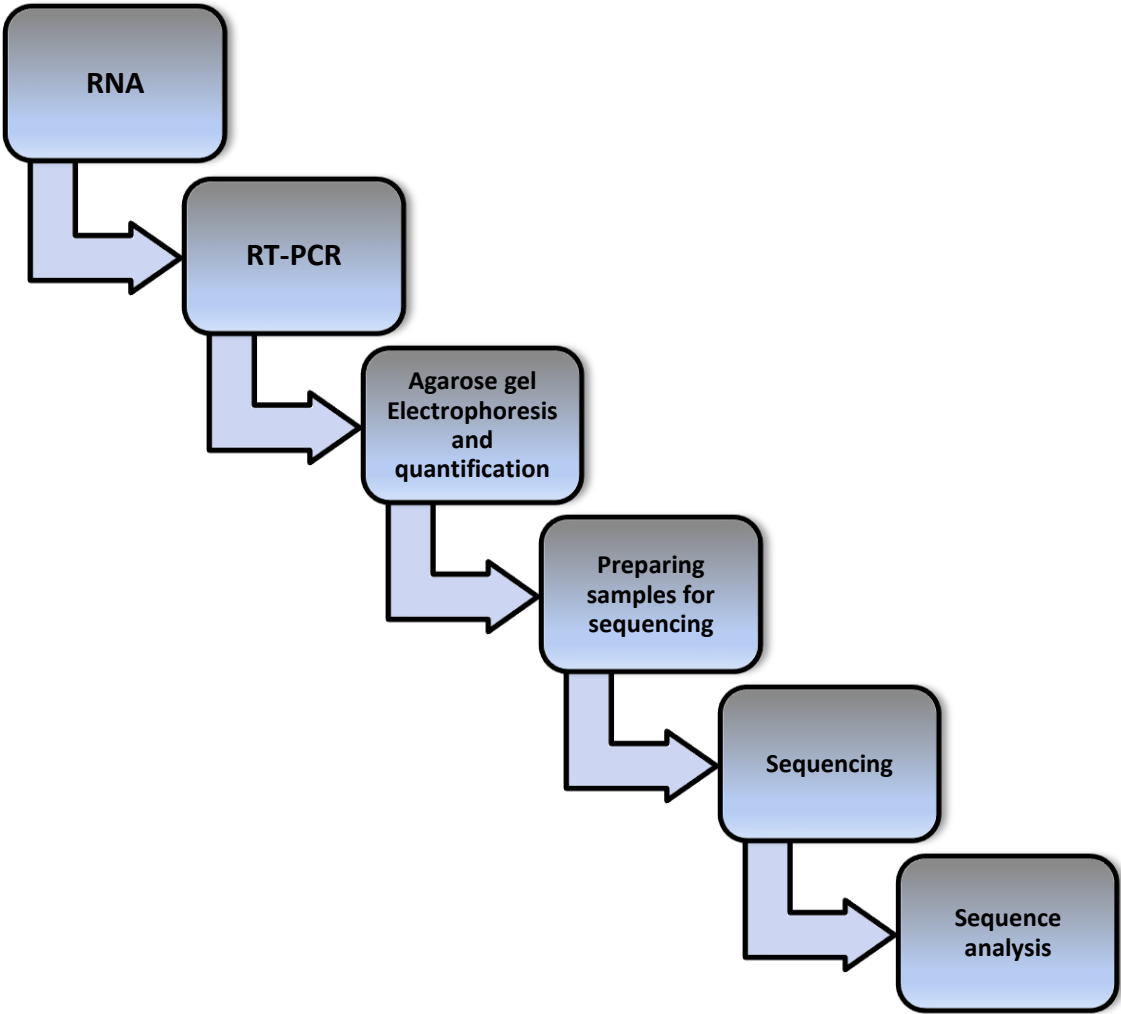

Supplement: S3 File — (PDF) [file pone.0262170.s003.pdf]
